# The moonlighting RNA-binding activity of cytosolic serine hydroxymethyltransferase contributes to control compartmentalization of serine metabolism

**DOI:** 10.1093/nar/gkz129

**Published:** 2019-02-27

**Authors:** Giulia Guiducci, Alessio Paone, Angela Tramonti, Giorgio Giardina, Serena Rinaldo, Amani Bouzidi, Maria C Magnifico, Marina Marani, Javier A Menendez, Alessandro Fatica, Alberto Macone, Alexandros Armaos, Gian G Tartaglia, Roberto Contestabile, Alessandro Paiardini, Francesca Cutruzzolà

**Affiliations:** 1 Department of Biochemical Sciences, Sapienza University of Rome - P. le Aldo Moro 5, 00185 Rome, Italy; 2 Istituto di Biologia e Patologia Molecolari, Consiglio Nazionale delle Ricerche, 00185 Rome, Italy; 3 Program Against Cancer Therapeutic Resistance (ProCURE), Metabolism and Cancer Group, Catalan Institute of Oncology, 17007 Girona, Catalonia, Spain; 4 Molecular Oncology Group, Girona Biomedical Research Institute (IDIBGI), 17190 Girona, Spain; 5 Department of Biology and Biotechnology ‘C. Darwin', Sapienza University of Rome, 00185 Rome, Italy; 6 Centre for Genomic Regulation (CRG), The Barcelona Institute for Science and Technology, Dr. Aiguader 88, 08003 Barcelona, Spain; 7 Universitat Pompeu Fabra (UPF), Department of Experimental and Health Sciences, 08003 Barcelona, Spain; 8 Institucio Catalana de Recerca i Estudis Avançats (ICREA), Department of Life and Medical Sciences, 23 Passeig Lluıs Companys, 08010 Barcelona, Spain

## Abstract

Enzymes of intermediary metabolism are often reported to have moonlighting functions as RNA-binding proteins and have regulatory roles beyond their primary activities. Human serine hydroxymethyltransferase (SHMT) is essential for the one-carbon metabolism, which sustains growth and proliferation in normal and tumour cells. Here, we characterize the RNA-binding function of cytosolic SHMT (SHMT1) *in vitro* and using cancer cell models. We show that SHMT1 controls the expression of its mitochondrial counterpart (SHMT2) by binding to the 5′untranslated region of the SHMT2 transcript (UTR2). Importantly, binding to RNA is modulated by metabolites *in vitro* and the formation of the SHMT1–UTR2 complex inhibits the serine cleavage activity of the SHMT1, without affecting the reverse reaction. Transfection of UTR2 in cancer cells controls SHMT1 activity and reduces cell viability. We propose a novel mechanism of SHMT regulation, which interconnects RNA and metabolites levels to control the cross-talk between cytosolic and mitochondrial compartments of serine metabolism.

## INTRODUCTION

Many eukaryotic enzymes belonging to the intermediary metabolism have been shown to perform other functions in addition to their primary metabolic role (so-called moonlighting proteins). A large number of these metabolic enzymes are able to function as RNA-binding proteins and play crucial roles in post-transcriptional gene regulation and in the control of activity and localization of enzymatic complexes (1). Examples of such moonlighting enzymes include thymidylate synthase (TYMS), dihydrofolate reductase (DHFR) and glyceraldehyde 3-phosphate dehydrogenase (GAPDH) (2–4).

Human serine hydroxymethyltransferase (SHMT) catalyses the reversible conversion of serine and tetrahydrofolate (THF) into glycine and 5,10-methylenetetrahydrofolate (CH_2_-THF). This reaction is central in the serine-glycine one-carbon metabolism (1C-metabolism), a complex network fuelling the biosynthesis of nucleotide precursors, NADPH and methylation factors and thus sustaining cell growth and proliferation. The human genome contains two *shmt* genes, encoding three main SHMT isoforms that differ in sequence and/or localization: one mitochondrial isoform, named SHMT2, and two cytosolic isoforms, SHMT1 and SHMT2α, the latter lacking the mitochondrial import signal present in SHMT2 and thus localizing in the cytosol.

The mitochondrial SHMT2 isozyme is mainly involved in the serine-derived production of both glycine and one-carbon units necessary to fuel the synthesis of purines, mitochondrial thymidine monophosphate (dTMP) and antioxidant molecules such as NADPH and glutathione. SHMT2 is also required for the maintenance of transfer RNA (tRNA) pools inside the mitochondria, affecting the levels of formylmethionyl-tRNA and other methylated tRNAs, and thereby controlling the translation of mitochondrial respiratory complexes (5–8). By contrast, SHMT1 is likely to have a more versatile metabolic role, since it can switch the directionality of the reaction depending on cell type and metabolic needs (5,9). In addition, SHMT1, together with SHMT2α, undergoes nuclear localization during the S-phase of the cell cycle to participate in the synthesis of dTMP (10,11).

The reaction catalyzed by SHMT is pivotal for the metabolic reprogramming of cancer cells and, not surprisingly, tumours often overexpress one or more isoforms (12). SHMT1 and SHMT2 are up-regulated in patient-derived lung cancer tissue samples (13,14). We previously showed that SHMT1 knockdown in A549 and H1299 lung cancer cell lines triggered apoptosis and induced a compensatory increase of SHMT2α expression by a yet unknown mechanism, suggesting that SHMT1 might be involved in the regulation of the other isoforms (13). Since the SHMT1 can bind RNA (15), we have hypothesized that the reported regulation could occur at the post-transcriptional level. There are many examples of regulatory proteins and enzymes interacting with the 5′untranslated regions (5′UTRs) of their target transcripts to modulate the stability and the translation of messenger RNAs (mRNAs) (16), including TYMS and DHFR (2,3,17).

We therefore investigated whether SHMT1 could regulate the expression of the other isozymes by binding to their transcripts via 5′UTR recognition. Here, we characterized the binding of SHMT1 to the 5′UTR of its mRNA and to three 5′UTRs of SHMT2 isoforms differently expressed in lung cancer, chosen on the basis of their relative abundance in RNA-sequencing data (18). Our data demonstrate that SHMT1 binds specifically and with high affinity to the 5′UTR of SHMT2, affecting the expression and the translation of the corresponding transcript. The SHMT1/RNA interaction is modulated by the enzyme’s substrates. Unexpectedly, binding of SHMT1 to the RNA moiety selectively inhibits the SHMT1 enzymatic activity, since the cleavage of serine to glycine is significantly more affected than the opposite reaction (i.e. glycine to serine). Our results show that the RNA-mediated inhibition is also effective in cancer cell lines, suggesting that it may contribute to control serine consumption by the cytosolic SHMT1.

In summary, our work allow us to assign a biologically relevant role to the moonlighting RNA-binding activity of SHMT1 (19) and to propose a novel regulatory mechanism involving SHMT1, RNA species and metabolites important to fine-tune the interplay between cytosolic and mitochondrial isozymes.

## MATERIALS AND METHODS

### Materials

Chemicals and reagents 2-(4-chlorophenyl)ethylamine, ethyl chloroformate (ECF), diethyl ether, ethyl acetate, dichloromethane and thymidine 5′-monophosphate disodium salt were obtained from Sigma-Aldrich. Tetrahydrofolate and (6S)-5-formyl tetrahydrofolate were kindly provided by Merck & Cie (Schaffhausen, Switzerland). Tritiated glycine used in the radioisotopic assay ([2−3H]glycine) was purchased from Perkin Elmer. (6R,S)-5-Formyl-5,6,7,8-tetrahydropteroylpenta-γ-L-glutamic acid lithium salt (5-CHO-THF-Glu_5_) was purchased from Schircks Laboratories, Bauma, Switzerland. All other reagents used in the enzymatic assays were obtained from Sigma-Aldrich.

### Protein expression and purification

Wild-type and mutant SHMT genes were cloned into a pET22b(+) vector (Novagen) and expressed as N-terminal Histidine-tagged fused proteins in *Escherichia coli* (BL21-DE3). Purification was performed as described in Giardina *et al.* (20). Briefly: cells were sonicated, and the soluble proteins were purified by affinity chromatography on a 5 ml HP-chelating column (GE Healthcare) loaded with nickel ions. Protein was eluted with 0.3 M imidazole. Protein buffer was then exchanged on desalting columns PD10 (GE Healthcare) and the histidine tag removed by proteolytic digestion with 1U/mg of thrombin (SIGMA) at 16°C for 15 h. The digestion mix was then loaded again on the HP-chelating column and thrombin eluted in the flow thought, whereas SHMT, which binds with low affinity, was eluted with 0.1 M imidazole. The proteins were then concentrated and injected into a Superdex 200 column (either 10/300 or 16/60; GE Healthcare) and eluted with the following buffer; 20 mM 4(-2-hydroxyethyl)-1-piperazineethanesulfonic acid (HEPES) pH 7.4, 5% glycerol, 100 mM NaCl (for SHMT1 and Y82A) or 200 mM NaCl (for K157S-K158S), flash freezed in liquid nitrogen and stored at −20°C. All proteins resulted mainly tetrameric in solution; the K157S-K158S mutant required the addition of 200 μM pyridoxal phosphate (PLP) to the running buffer to fully populate the tetrameric state, whereas in the absence of PLP it eluted as a mixture of tetramer (55%) and dimer (45%) (Supplementary Figure S4A). The dichroic spectra of the two mutants superpose with the wild-type enzyme and their thermal stability is slightly reduced (Supplementary Figure S4B-C), as observed for other mutations located near the active site or the tetrameric interface (11).

### *In vitro* transcription

pGL3 plasmids (pGL3_Control) containing the specific UTRs cloned upstream of the luciferase gene were provided by GeneArt/Invitrogen (Ratisbonne, Germany).

UTR sequences were amplified by polymerase chain reaction (PCR) from pGL3 plasmids using primers designed to include the T7 promoter sequence upstream of the UTR PCR fragment (primers listed in Supplementary Table S1). PCR products were purified from agarose gel using the Nucleospin Gel and PCR Clean-up Kit (Macherey-Nagel). The DNA fragments generated by PCR were used as templates to produce the corresponding RNA sequences with Ribomax Large Scale RNA Production System-T7 Kit (Promega).

### Biotinylation of RNA fragments

The UTR sequences were labelled at the 3′ end with a molecule of cytidine-5′-phosphate-3′-(6-aminohexyl)phosphate labelled with biotin (pCp-biotin) using the Pierce™ RNA 3′ End Biotinylation Kit (Thermo Scientific) and purified according to manufacturer instructions.

### Electrophoretic mobility shift assay

Gel mobility shift assays were conducted by incubating a fixed amount of RNA with fixed or increasing concentrations of purified human SHMT1. For competition assays increasing concentrations of unlabelled competitors UTRs were added to the reaction mixture. To determine the effect of metabolites upon RNA binding, the enzyme was pre-incubated with an excess of serine or glycine and 5-CHO-THF-Glu_n_ for 5 min at room temperature (RT), the latter being a substrate analogue stable in an oxidative environment. Electrophoretic mobility shift assays (EMSA) on the K157S/K158S mutant were run both in the absence (Figure 6D) and presence of PLP (data not shown), obtaining a similar binding profile, thus excluding that the dimeric aggregation state, partially populated in this mutant in absence of PLP (Supplementary Figure S4A), could hamper RNA binding.

All the components were incubated at RT for 30 min in 12 μl of binding buffer (20 mM HEPES pH 7.4, 100 mM NaCl) containing 20 μg/ml bovine serum albumin (BSA), 1 μg of tRNA, 1 U/μl of recombinant Rnasin ribonuclease Inhibitor and 8% (v/v) glycerol. The reaction mixtures were separated onto 4% nondenaturing polyacrylamide gels in 0.5× TBE buffer (45 mM Tris-Borate, 1 mM ethylenediaminetetraacetic acid, pH 8.6). For the visualization of unlabelled sequences, gels were stained with SYBR Safe (Invitrogen) in 30 ml of 0.5× TBE. To detect biotin-labelled RNA, gels were transferred onto Immobilon-Ny+ positive charged nylon membranes (0.45 μm) in 0.5× TBE, the RNA was then cross-linked to the membrane using a hand-held UV lamp equipped with 314 nm bulbs. The biotin moiety was detected by chemiluminescence using the Chemiluminescent Nucleic Acid Detection Module (Thermo Scientific). In both experiments, images were acquired using Chemidoc MP Imaging System (Bio-Rad). All experiments were run in triplicate. Densitometric measurements of the bands corresponding to free RNA molecules were transformed into percentages, the amount of bound RNA (% bound) was then calculated by subtracting the percentage of free RNA from the total, the result was plotted as a function of protein concentration. The apparent dissociation constants (*K*_d_^app^) were estimated fitting the data with Equation (1), in which *B*_max_ corresponds to the maximum binding (100%) and [P] corresponds to the concentration of SHMT1 in the reaction mixture.

(1) \begin{equation*}\% \ {\rm Bound}\ = \ \frac{{{B_{{\rm max}}}\left[ P \right]}}{{{K_{\rm d}} + \left[ P \right]}}\end{equation*}

### Site-directed mutagenesis

SHMT mutants were produced by site-directed mutagenesis using the QuikChange Lightning Site-Directed Mutagenesis Kit (Agilent Technologies). The pET28b containing the wild-type SHMT1 was used as template DNA. Two complementary oligonucleotides, synthesized by Metabion (Steinkirchen, Germany), were used as primers for the mutagenesis reactions (primers listed in Supplementary Table S1). *Escherichia coli* Neb5α cells were transformed to amplify the mutated plasmid and mutagenesis was verified by sequencing.

### Cell lines

H1299 cancer cells were grown in RPMI-1640 medium supplemented with 2 mM L-glutamine, 100 IU/ml penicillin/streptomycin and 10% fetal bovine serum (FBS; Biowest USA). HAP1 and HAP1shmt2KO cells were obtained from Horizon Discovery Ltd., Cambridge, UK and maintained in IMDM medium (Gibco) supplemented with 100 IU/ml penicillin/streptomycin and 10% FBS (Biowest USA). Minimal medium (MEM) formulation and MEM supplementations have been previously reported (21).

### Cells transfection

H1299 and HAP1-SHMT2KO cells were transiently transfected with interference RNA sequences from Qiagen as previously reported (13) using JetPrime (Polyplus transfection). For the rescue experiments, at 24 h after the transfection, the medium was replaced with fresh medium with the specified supplementation.

### RNA extraction and real-time qRT-PCR analysis

Total RNA was extracted from cells using *TRIzol* reagent (Invitrogen) following the manufacturer instructions. Reverse transcription reactions were performed on 1 μg of total RNA with *SuperScript First-Strand Synthesis System Kit* (Invitrogen). Each PCR tube contained 5 μl 2 × *KAPA Sybr Fast universal* premix, 2 × 0.4 μl primer, 1 μl complementary DNA (1:5 diluted) and 3.2 μl water (final volume 10 μl). Reactions were performed using Stratagene MX3000P (Stratagene, La Jolla, CA, USA). The cycling protocol was: 95°C for 2 min, 40 cycles of 95°C for 5 s, 60°C for 25 s, before fluorescence detection. A melting curve was determined using the standard instrument protocol. Amplification specificity was assessed by analysis of melting curves. Relative fold change of the expression of individual genes was calculated using the 2^−ΔΔCt^. Each sample was analysed in triplicate. Primer sequences are shown in Supplementary Table S1.

### Western blotting

Cells were lysed in 8 M urea (Sigma-Aldrich) and protein concentration was determined using the Biorad Protein Assay (Bio-Rad, Munchen, Germany). Protein samples (40 μg) were subjected to Sodium dodecyl sulphate-polyacrylamide gel electrophoresis, transferred onto nitrocellulose membranes and then saturated with 5% BSA in Tris-buffered saline with 0.1% Tween 20. Membranes were then incubated with the primary antibody for 18  h at 4°C and subsequently with horseradish peroxidase-conjugated secondary antibody for 1 h at RT. Membranes were washed with Tris-buffered saline in 0.1% Tween 20 and developed using the chemiluminescence system Chemidoc MP Imaging System (Bio-Rad). The anti-SHMT2, -β-actin and the anti-goat and -mouse antibody were from Santa Cruz Biotechnology (Santa Cruz, CA, USA); the anti-SHMT1 antibody was from Cell Signaling Technology (Danvers, Massachusetts, USA).

### Luciferase assay

Luciferase activity was determined after transfecting the pGL3 plasmids into the H1299 cells. Cells were co-transfected with 0.5 μg *Renilla* luciferase as control of transfection together with 1 μg of the pGL3, pGL3-UTR2 or pGL3-UTR1 plasmid plus iscr or the specific interference RNA (iRNA) against *shmt1*. The expression of Firefly and Renilla luciferases was analysed 48 h after transfection using the Dual-Luciferase® Reporter Assay System (Promega) in a Victor3™ multilabel counter (Perkin Elmer, Waltham, Massachusetts, USA). Firefly luciferase was standardized to the value of Renilla.

### Trypan blue exclusion assay

The growth medium was collected and the cells were washed once with phosphate buffered saline (PBS) (also collected). Adherent cells were removed by treatment with trypsin, which in turn was blocked using complete medium. All the collected cell fractions were centrifuged 5 min at 1000 × *g* and the supernatant carefully discarded. The harvested cells were then washed with PBS, centrifuged 5 min at 1000 × *g* and the supernatant discarded. Following the addition of a 0.4% (w/v) trypan blue solution (Sigma-Aldrich) cells were transferred to a Burker counting chamber (Hirschmann, Germany) and visualized using an Axioskop 2 plus microscope (Carl Zeiss Microscopy, Switzerland). Cells stained with trypan blue dye were considered nonviable.

### *In vitro* enzymatic activity assays

All assays were carried out at 30°C in 20 mM KPi buffer at pH 7.2, treated with diethyl pyrocarbonate (DEPC). The SHMT activity was measured using 0.2 μM enzyme with L-serine and THF as substrates by means of a spectrophotometric coupled assay, in which the 5,10-CH_2_-THF produced by the reaction was oxidized by the NAD-dependent *E. coli* 5,10-CH_2_-tetrahydrofolate dehydrogenase (22). The possible inhibition of methylenetetrahydrofolate dehydrogenase by RNA was checked in an experiment in which the activity of the enzyme (1 μM) with 50 μM 5,10-CH_2_-THF and 250 μM NADP^+^ was measured in the presence and absence of tRNA and UTR2. No inhibition was observed under these conditions.

Two assays were used to analyse the inverse reaction. The first assay uses glycine and 5-CHO-THF as substrates and is based on the spectrophotometric measurement of the quinonoid intermediate that develops when both ligands bind to SHMT, forming an enzyme–glycine–folate ternary complex. The quinonoid intermediate, which yields an intense absorption band with a maximum at 502 nm, derives from deprotonation of glycine, but it accumulates to a measurable extent only when a folate ligand is also bound to SHMT and a ternary complex is formed (23). Therefore, the absorbance at 502 nm is proportional to the fraction of enzyme present as the ternary complex. The second assay is a coupled assay carried out in the presence of 5,10-CH_2_-THF and glycine substrates. The L-serine product of this reaction is oxidized by a NAD^+^-dependent serine dehydrogenase.

The inhibition curves were obtained keeping fixed concentration of substrates (10 mM glycine, 10 mM L-serine, 5 μM 5-CHO-THF and 80 μM THF, 90 μM 5,10-CH_2_-THF) while varying the RNA concentration. All obtained inhibition curves were fitted to Equation (2), in which [I] corresponds to the RNA concentration.

(2) \begin{equation*}\% {\rm Activity}\ = \ 100\left( {1 - \frac{{\left[ I \right]}}{{\left[ I \right] + I{C_{50}}}}} \right)\end{equation*}

The characterization of the tRNA inhibition mechanism was carried out using serine and THF as substrates. In a first series of experiments, L-serine concentration was held constant at 10 mM, while THF was varied between 10 and 488 μM. The single saturation curves obtained varying THF at various RNA concentrations were fitted to a modified Michaelis–Menten equation that accounts for substrate inhibition (Equation 3).

(3) \begin{equation*}{v_{\rm i}} = {v_{{\rm max}}}\ \frac{{\left[ {{\rm THF}} \right]}}{{{K_{\rm m}} + \left[ {{\rm THF}} \right]\left( {1 + \frac{{\left[ {{\rm THF}} \right]}}{{K{{\rm i}_{{\rm THF}}}}}} \right)}}\end{equation*}

Moreover, all the saturation curves were globally fitted to a modified Michaelis–Menten equation that accounts for both substrate inhibition and hyperbolic competitive inhibition (Equation 4).

(4) \begin{equation*}{v_{\rm i}} = {v_{{\rm max}}}\ \frac{{\left[ {{\rm THF}} \right]}}{{{K_{\rm m}}\left( {\frac{{1 + \frac{{\left[ {{\rm tRNA}} \right]}}{{{K_{\rm i}}}}}}{{1 + \frac{{\left[ {{\rm tRNA}} \right]}}{{\alpha {K_{\rm i}}}}}}} \right) + \left[ {{\rm THF}} \right]\left( {1 + \frac{{\left[ {{\rm THF}} \right]}}{{K{{\rm i}_{{\rm THF}}}}}} \right)}}\end{equation*}

In these equations, *v*_i_ stands for initial velocity, *V*_max_ is the maximum velocity, *K*_m_ is the Michaelis–Menten constant for THF, *K*i_THF_is the substrate inhibition constant, *K*i is the RNA inhibition constant and *α* is the factor by which *K*i changes when THF is bound to the enzyme.

In a second series of experiments, THF concentration was held constant at 80 mM, while L-serine was varied between 0.156 and 10 mM. The saturation curves obtained were fitted to a Michaelis–Menten equation.

All spectrophotometric measurements were performed using a Hewlett-Packard 8453 diode-array spectrophotometer. Fitting of data to equations was carried out with the PRISM software (GraphPad, La Jolla, CA, USA).

### SHMT activity assay in living cells

SHMT activity measurements in living cells were performed using a radioisotope assay based on the capability of SHMT to catalyze the exchange of the pro-2S proton of glycine with solvent (24,25). At 24 h post-transfection, H1299 cells were detached using trypsin and washed twice in 2 ml of PBS buffer followed by centrifugation to eliminate the growth medium. Aliquots (100 μl) of cells suspension were incubated with [2−3H]glycine (2 × 109 dpm μmol^−1^) (23 μM) for 4 h at 37°C. Subsequently, samples were centrifuged to remove cells and reactions were stopped by the addition of 3% (w/v) trichloroacetic acid to remove radiolabelled glycine and measure radioactivity in the solvent. Control reactions were performed to correct for background exchange (these samples did not contain cells). All measurements were performed in duplicate and were normalized on the basis of protein concentration.

### Serine consumption assay

Sample extraction and derivatization. Serine was analysed according to the method of Gao *et al.* (26) with slight modifications. Cells transfected as indicated were cultured in MEM originally supplemented as described before and specifically with 400 μM serine. After 48 h, cells were harvested and centrifuged to eliminate cellular debris. Then, 450 μl cell culture medium was spiked with 20 μl of 0.7 mM 2-(4-chlorophenyl)ethylamine as an internal standard. The first step of derivatization was performed by adding 350 μl of ethanol/pyridine (6:1) and 50 μl of ECF. The reaction mixture was then vortexed for 30 s and ultrasonicated for 60 s to increase the speed of reaction at RT. The derivatization products were extracted with 700 μl of n-hexane for 60 s and centrifuged for 5 min at 3000 rpm. The organic layer was removed and the aqueous phase was adjusted to pH ≥12 with 0.1 ml 7M NaOH. The second derivatization step was performed by adding a further 50 μl of ECF and 700 μl of n-hexane. The reaction mixture was again vortexed and ultrasonicated as before, and then centrifuged at 3000 rpm for 10 min. The organic layers were combined, dried under N2, resuspended in dichloromethane and analysed by GC/MS gascromatography-mass spectrometry (GC/MS).

GC-M analysis was performed using an Agilent 6850A gas chromatograph coupled to a 5973N quadrupole mass selective detector (Agilent Technologies, Palo Alto, CA, USA). Chromatographic separations were carried out with an Agilent HP5ms fused-silica capillary column (30 m × 0.25 mm i.d.) coated with 5%- phenyl-95%-dimethylpolysiloxane (film thickness 0.25 μm) as stationary phase as follows: injection mode: splitless at a temperature of 280°C; column temperature program: 80°C (2 min) ramped to 140°C at a rate of 10°C/min, to 240°C at a rate of 4°C/min, to 280°C at a rate of 10°C/min and held for 5 min. The carrier gas was helium at a constant flow of 1.0 ml/min. The spectra were obtained in the electron impact mode at 70 eV ionization energy; ion source 280°C; ion source vacuum 10^−5^ Torr.

MS analysis was performed simultaneously in TIC (mass range scan from 50 to 600 m/z at a rate of 0.42 scans/s) and SIM mode. GC-SIM-MS analysis was performed selecting the following ions: 102 m/z for glycine, 114 m/z for serine and 227 m/z for 2-(4-chlorophenyl)ethylamine (internal standard).

### Protein–RNA interaction predictions

We retrieved all the 5′UTR sequences available from the Ensembl database (27) version GRCh38.p12. We restricted our analysis to 164 sequences having the same length as the SHMT2 5′UTR (UTR2) sequence and used *Global Score* (28) and *omiXcore* (29) to predict their binding potential and their binding affinity (Supplementary Table S2). *Global* Score distinguishes interacting from non-interacting RNA-protein pairs, while *omiXcore* predicts the strength in the range [0,1] where values equal to zero suggest no interaction and values greater than zero represent the actual predicted binding affinity.

### Statistical analysis

Data are the mean ± standard deviation of at least three independent biological experiments, each repeated in three technical replicates. Paired samples of real time PCR data were analysed with Student’s *t*-test; all the others statistical analysis were performed using one way ANOVA followed by the Bonferroni post-hoc comparison test. A *P* < 0.05 was considered significant.

## RESULTS

### SHMT1 binds to the 5′UTR sequences of SHMT2 and SHMT2α

**Figure 1.**
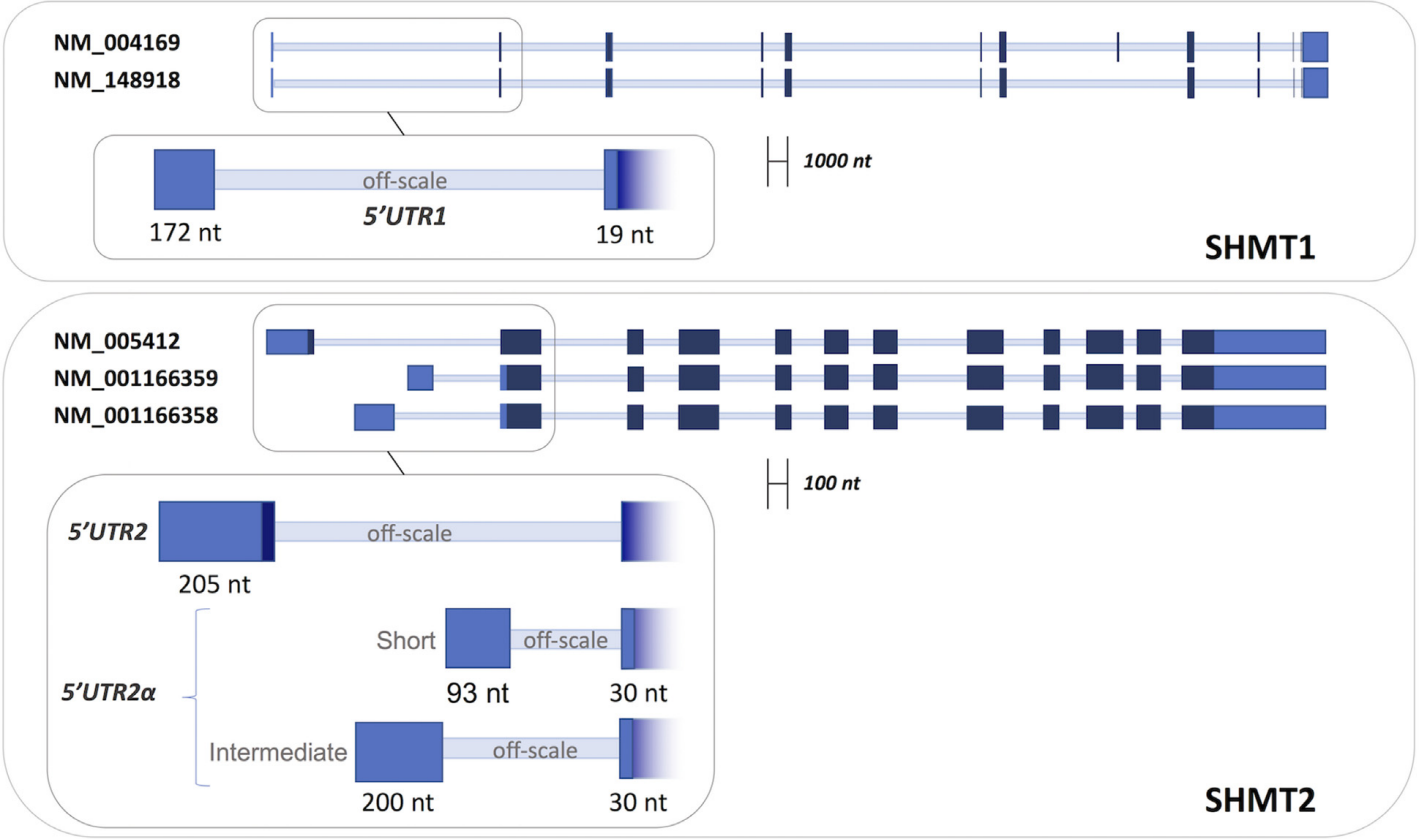
Schematic representation of the pre-mRNAs of different SHMT1 and SHMT2 transcripts. Non-coding exons are colored in light blue, coding exons in dark blue and introns are represented as thin light blue lines. The 5′UTR sequences selected for the experiments, composed by the indicated exons, are shown in the inset. **UTR1** = (NM_004169-NM148918: Ex1_21–192+Ex2_1–19). **UTR2** = (NM005412:Ex1_1–205). **UTR2αsh** = (NM_001166359: Ex1_1–93+Ex2_1–30). **UTR2αint** = (NM_001166358: Ex1_1–200+Ex2_1–30).

**Figure 2.**
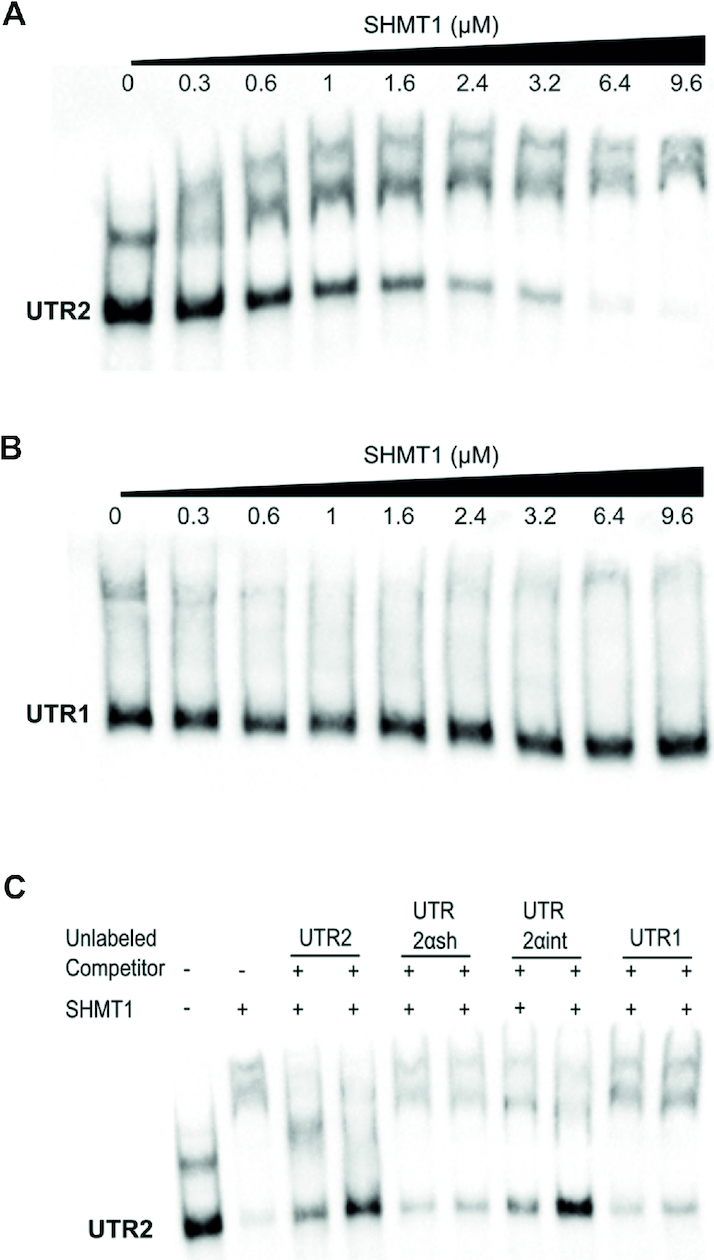
Electrophoretic mobility shift assay. The first two panels show the migration of 0.08 μM biotin-labelled UTR2 (**A**) and UTR1 (**B**) alone or in the presence of the indicated concentrations of SHMT1 (3.75–125 fold excess SHMT1:RNA). (**C**) Competition assay performed by incubating 0.08 μM of biotin-labelled UTR2 with 4.8 μM SHMT1 and, respectively, 0.6 and 1.2 μM of unlabelled competitors (UTR2, UTR2αsh, UTR2αint and UTR1).

SHMT1 and SHMT2 expression levels are cross-related in lung cancer cells (11,13); however, the mechanisms by which their expression is controlled to ensure the appropriate amounts of each isoform are unknown. We hypothesized that the control may involve the interaction of SHMT1 with the 5′UTR region of the SHMT2 transcripts, since it has been shown that SHMT1 can bind its own RNA at the level of 5′UTR. Analysis of the expression of SHMT transcripts in samples of lung adenocarcinoma (LUAD) cell lines, obtained from the TCGA database (18), allowed us to identify several transcripts arising from the *shmt2* gene, together with those from the *shmt1* gene (Supplementary Table S3). To assess the capability of the SHMT1 protein to bind the 5′UTR of these transcripts *in vitro*, three SHMT2 RNAs, found to be expressed at different levels (more than 90%, i.e. NM_005412; below 5%, i.e. NM_001166358, and below 0.1% i.e. NM_001166359), and two SHMT1 transcripts sharing the same 5′UTR (i.e. NM_004169 and NM_148918) were chosen. The features of the 5′UTR fragments employed in the SHMT1-binding experiments, respectively named UTR2, UTR2αint, UTR2αsh and UTR1, are shown in Figure 1 (complete nucleotide sequences reported in Supplementary Table S4) together with the pre-mRNA structure of the corresponding transcripts. To assess whether SHMT1 binds to the selected RNA species, we performed EMSA. Results showed that migration of the UTR2 (Figure 2A and Supplementary Figure S1D), UTR2αint and UTR2αsh RNA fragments (Supplementary Figure S1A,B) was retarded in the presence of increasing concentrations of SHMT1 (0.3–9.6 μM), indicating the formation of RNA–protein complexes. SHMT1 was unable to alter the migration of its own 5′UTR fragment (UTR1) suggesting that the enzyme does not bind to this sequence under the same experimental conditions and concentration range (Figure 2B and Supplementary Figure S1C); indeed, previous data indicate that SHMT1 can bind UTR1 (15), but only at a very high SHMT1:RNA molar ratio (25000:1), which is far higher than the more stringent conditions used in the present study.

To assess which 5′UTR sequence binds with higher relative propensity, a competition assay was performed (Figure 2C). Biotinylated UTR2 was incubated with SHMT1 in the presence of two different concentrations of each unlabelled UTR sequence. The fragments that displaced the bound UTR2 RNA are UTR2 itself and UTR2αint, whereas, as expected from the experiments shown above, UTR1 did not compete in the concentration range used. Interestingly, the UTR2αsh, which binds to the protein when assayed alone (Supplementary Figure S1B), did not significantly compete with the UTR2–protein complex.

**Figure 3.**
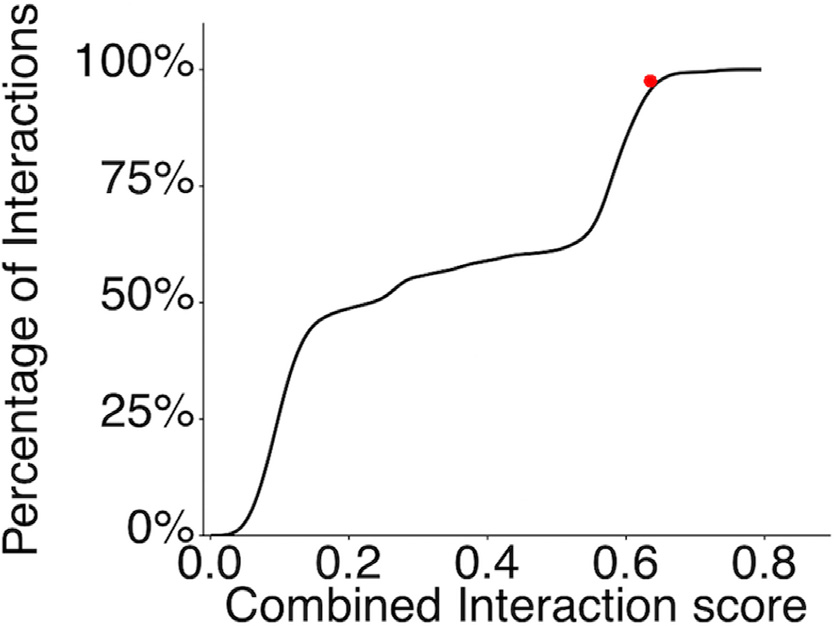
*Global Score* and *omiXcore* scores were linearly combined in the Combined Interaction Score to estimate the binding preference of SHMT1 to UTR2. The cumulative distribution function of the Combined Interaction Score of SHMT1 against 164 5′UTRs sequences with length of 206 nts is shown. The interaction affinity of SHMT1 against UTR2 is marked in red (top 2% of all cases).

An initial computational analysis, based on the *Global Score* algorithm (28), predicted both UTR2 and UTR2αint as interactors (both have the maximum score of 1, while the control RNA has a score of 0). Using *omiXcore* to better evaluate the binding strength (29), only UTR2 scored above the significance threshold (UTR2: 0.27; threshold: 0.25; UTR2αint: 0.24; control: 0.14). Moreover, when combining *Global Score* and *omiXcore* analysis of SHMT1 interactions with a population of human 5′UTRs of the same length (164 cases) as UTR2 (206 nt; Figure 3 and ‘Materials and Methods’ section), UTR2 ranked in the top 2% of all cases, indicating a strong preference for binding.

**Figure 4.**
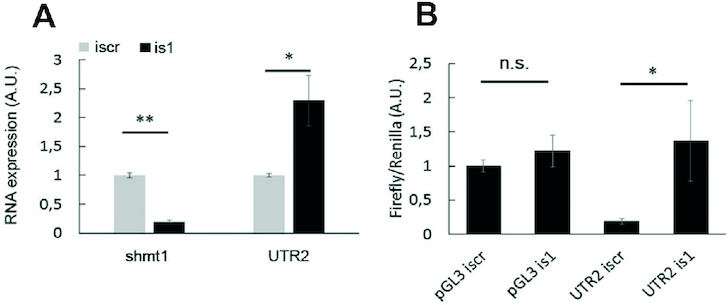
(**A**) Effect of SHMT1 on UTR2 levels in H1299 lung cancer cells. Expression of shmt1 and UTR2 RNAs, measured 48 h after transfection with the iscramble (iscr) or ishmt1 (is1). (**B**) Effect of SHMT1 on the translation of UTR2-containing luciferase reporter mRNA in H1299 lung cancer cells. The panel shows the ratio between firefly and Renilla luciferase evaluated 24 h after transfection with the indicated plasmids (empty pGL3 or pGL3 containing UTR2) and the indicated RNAis. Reduction of SHMT1 by interference increases the amount of translation from UTR2-containing transcripts (UTR2 is1) with respect to control (UTR2 iscr). Statistical analysis is performed on three independent experiments. * *P* ≤ 0.05 ** *P* ≤ 0.01

We next investigated whether the presence of the SHMT1 protein is able to modulate the levels of the SHMT2 transcripts in the cells. This analysis was focused on UTR2, which (i) is present in the most expressed transcript in lung cancer cells (Supplementary Table S3), (ii) shows optimal binding to SHMT1 *in vitro* and (iii) has an optimal interaction score, as predicted by computational means. The experiments were carried out in the LUAD H1299 cell line, a cellular model in which we previously observed the cross-talk between SHMT1 and SHMT2 (13). We transfected the cells with an RNA interference (RNAi) against *shmt1* (ishmt1) or a scramble RNA sequence (iscr) and then studied the expression of the UTR2 by qRT-PCR. As shown in Figure 4A, SHMT1 knockdown resulted in a significant increase of UTR2 levels. To verify if SHMT1 is able to affect the translation of UTR2-containing transcripts in the same cell line, a construct containing UTR2 cloned upstream of the firefly luciferase reporter gene in the pGL3 plasmid was employed. A significant increase (5-fold) in the luciferase signal was observed only in those cells co-transfected with the UTR2-containing plasmid and ishmt1 (Figure 4B). These experiments demonstrate that the presence of SHMT1 reduces the luciferase expression in the cells when the latter is controlled by UTR2, suggesting that SHMT1 is able to control translation of UTR2-containing transcripts. In line with this hypothesis, SHMT1 affects the *in vitro* translation of the UTR2-luciferase mRNA (Supplementary Figure S2).

### SHMT substrates modulate RNA binding

**Figure 5.**
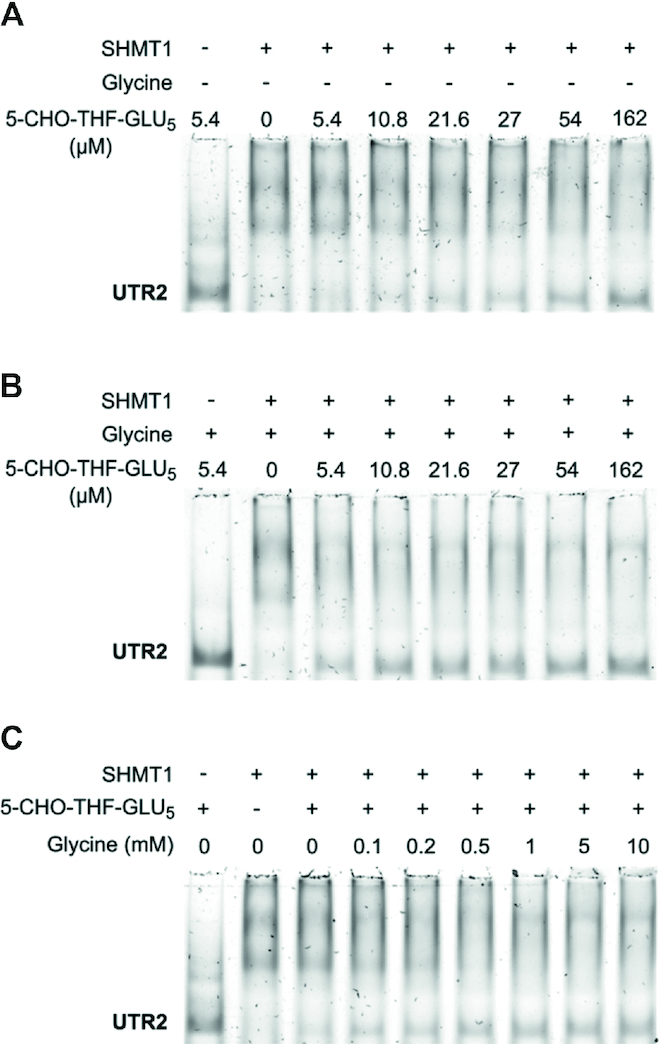
Effect of SHMT substrates on the binding of UTR2. EMSA carried out by incubating 5.4 μM of SHMT1 with the indicated amounts of 5-CHO-THF-Glu_5_ in absence (**A**) or presence (**B**) of 10 mM glycine prior to the addition of 0.18 μM unlabelled UTR2. (**C**) Effects of glycine concentration on the folate-mediated displacement of UTR2. EMSA was performed incubating 5.4 μM of SHMT1 with the indicated amounts of glycine and a fixed concentration of 5-CHO-THF-Glu_5_ (27 μM) prior to the addition of 0.18 μM unlabelled UTR2.

To assess if the binding of SHMT substrates (or analogues) interferes with the binding of RNA, EMSA assays were performed in the presence of such metabolites. SHMT1 was incubated with its amino acid and/or folate substrates prior to the addition of UTR2. As shown in Figure 5A, pentaglutamylated 5-formyl-tetrahydrofolate (5-CHO-THF-Glu_5_) competed with the formation of the RNA–protein complex. The presence of a saturating concentration of glycine in the reaction mixture significantly lowers the minimal folate concentration needed to compete with UTR2 (Figure 5B). This can readily be interpreted, as it is well known that the folate moiety has higher affinity for the enzyme–glycine complex than for the free enzyme (23). Interestingly, even at the highest folate concentration, the RNA moiety was not fully displaced. Crystallographic and solution studies on the rabbit SHMT1 (30,31) show that the polyglutamylated folate binding stoichiometry is 0.5 per subunit (2 per tetramer). Assuming that the RNA randomly binds to each monomer and considering that the folate-mediated displacement affects only two subunits, it is likely that at least 50% of UTR2 remains bound to the other sites, thus explaining our observations.

To further confirm the important role of glycine in the competition between folate and RNA, we varied the glycine concentration from 0.1 to 10 mM at fixed concentration of 5-CHO-THF-Glu_5_. As expected, the folate-mediated displacement of the RNA from its binding site depended on the glycine concentration, and reached a plateau at a concentration ≥0.5 mM (Figure 5C). Notably, in the absence of 5-CHO-THF-Glu_5_, saturating concentrations of glycine (or serine) did not interfere with the formation of the enzyme–RNA complex (Supplementary Figure S3A). These experiments were also performed using monoglutamylated 5-formyl-tetrahydrofolate (5-CHO-THF-Glu_1_). Interestingly, we did not observe any competition between this folate species and the RNA for binding to SHMT1 (Supplementary Figure S3B). Moreover, in this case the presence of saturating concentration of glycine only slightly affects the formation of SHMT1–UTR2 complex (Supplementary Figure S3C). These results suggest that the poly-glutamate tail is mainly responsible for the displacement of the RNA molecule, leading us to hypothesize that the UTR2 and 5-CHO-THF-Glu_5_ binding sites could partially overlap.

**Figure 6.**
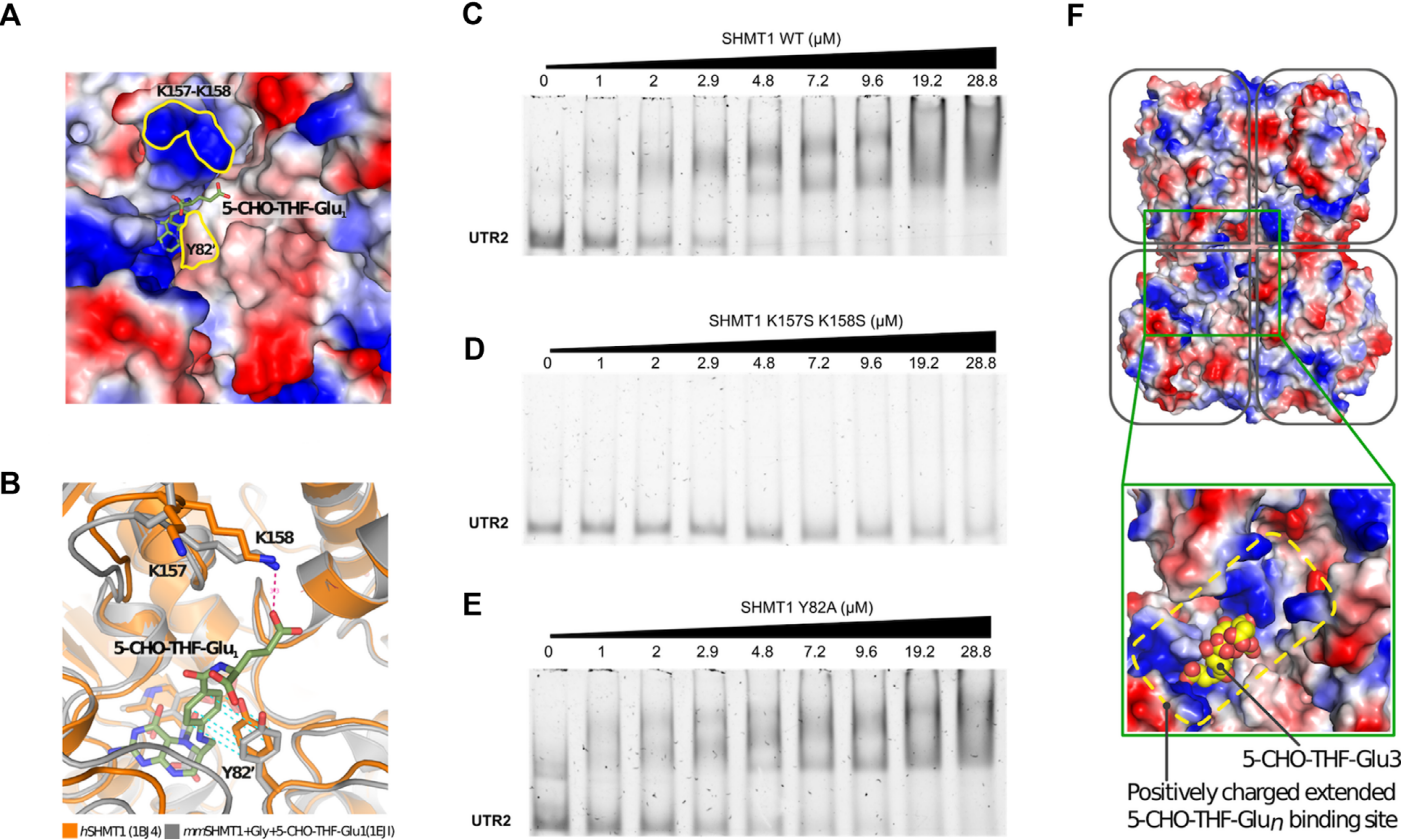
SHMT1 amino acid residues involved in folate and RNA binding. (**A**) Surface representation of the folate-binding site of human SHMT1 (*h*SHMT, PDB id: 1BJ4 (53)) coloured by electrostatic potential (red are acidic and blue basic residues). The substrate (5-CHO-THF-Glu_1_) from mouse SHMT1 (*mm*SHMT1, PDB id: 1EJI (54)) is shown as sticks and superposed to the human enzyme. The location of K157, K158 and Y82′ is highlighted by the yellow contours. (**B**) Superposition of the human and mouse crystal structures (in orange and grey, respectively) showing the interaction of K158 and Y82′ with the folate substrate. (**C**–**E**) EMSA assays carried out by incubating 0.24 μM of unlabelled UTR2 with the indicated concentrations of SHMT1 wild-type (WT) (C), K157S-K158S (D) and Y82A (E) mutants. The apparent dissociation constants (*K*_d_^app^) of the protein–UTR2 complexes were measured for the WT and for the Y82A mutant, and are respectively 1.39 ± 0.30 μM and 3.0 ± 0.68 μM. The *K*_d_^app^ value for the K157S-K158S SHMT1 mutant cannot be calculated given the low affinity of this protein for the UTR2 RNA in the concentration range explored. (**F**) Surface charge distribution of the human SHMT1 tetramer (PDB id: 1BJ4 (53)). In the blow up, 5-CHO-THF-Glu_3_, as observed in complex with rabbit SHMT1 (PDB id: 1LS3 (31)), is shown as yellow spheres superposed to the human enzyme. In the rabbit structure, the 5-CHO-THF-Glu_3_ is bound to two subunits (0.5 stoichiometry), with the poly-glutamylated tail making extensive and non-specific electrostatic interactions with the positively charged residues that surround the folate binding cleft.

It is known that formation of a protein–nucleic acids complex is often mediated not only by positively charged side chains but also by aromatic residues making stacking interactions with the nucleobases (32). In the structure of SHMT1, the folate-binding site is flanked by a loop harbouring two lysine residues (K157 and K158), one of which makes a salt bridge with the glutamic end of the folate. In addition, a tyrosine residue belonging to the other subunit (Y82′) forms an aromatic stacking interaction with the aminobenzoic moiety of folate (Figure 6A and B). To investigate whether these residues may play a role in UTR2–RNA binding, we characterized the behaviour of the K157S/K158S double and Y82A single mutants of SHMT1. As shown in Figure 6 (panels C–E), the lysine mutations almost completely abolished the binding of SHMT1 to UTR2, whereas, the Y82 substitution had a milder effect (2-fold) on the formation of the SHMT1–UTR2 complex. This further suggests that the folate and RNA-binding sites partially superimpose and are likely located in the positively charged region surrounding the folate binding cleft (Figure 6F).

### UTR2 binding affects the enzymatic activity of SHMT1

**Figure 7.**
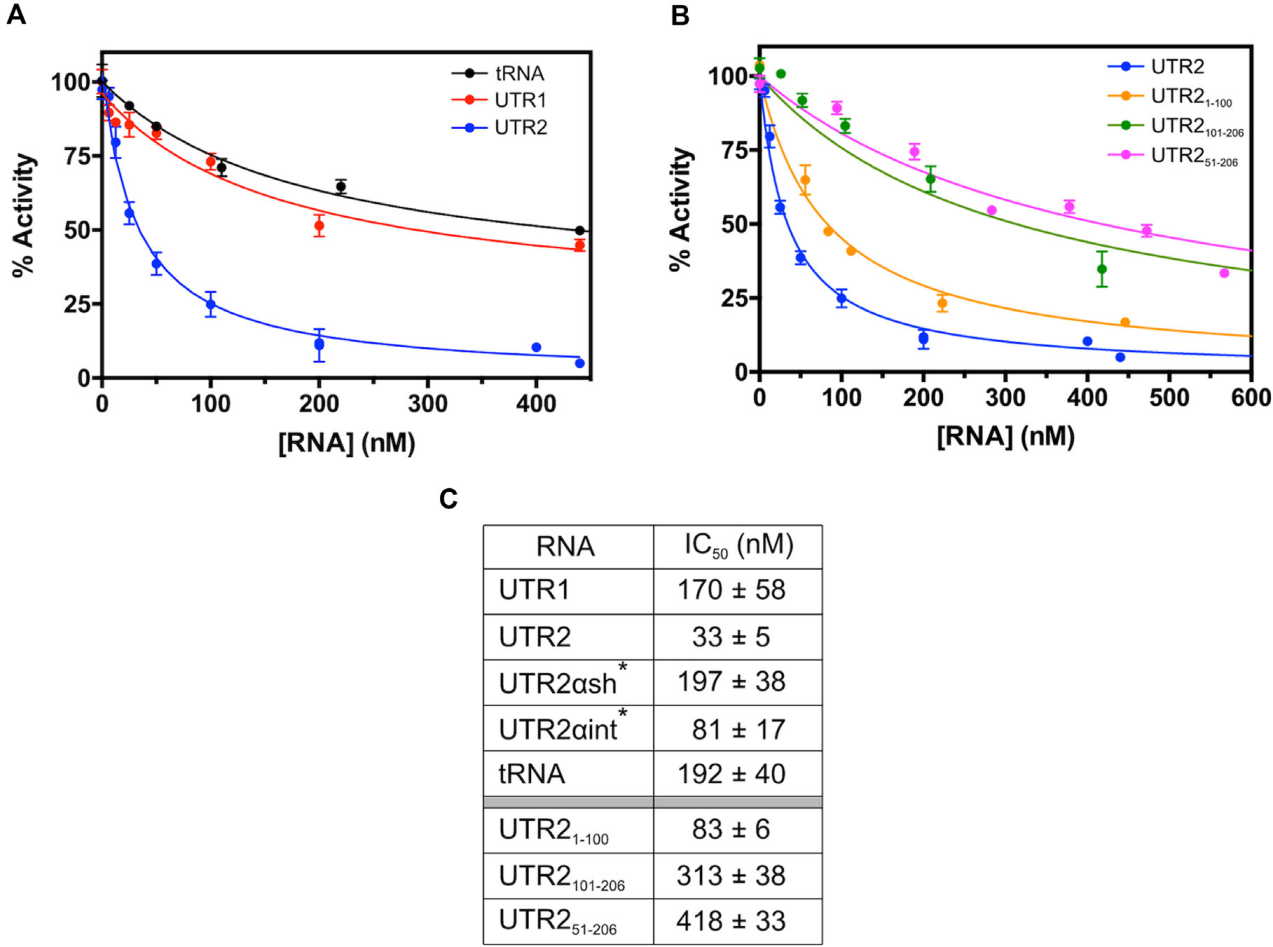
Inactivation of SHMT1 by RNA followed by measuring the initial velocity of the hydroxymethyltransferase reaction using L-serine and THF as substrates. (**A**) Inactivation of SHMT1 by three different RNAs, UTR1 (in red), UTR2 (in blue) and tRNA (in black) is reported. (**B**) Inactivation of SHMT1 by three UTR2 segments, namely UTR2_1–100_ (in orange), UTR2_101–206_ (in green) and UTR2_51–206_ (in pink). All the experimental data, acquired in three independent experiments, were fitted to Equation (2) (see ‘Materials and Methods’ section), obtaining the continuous lines shown in the figures; the corresponding IC_50_ values are reported in (C). *The experimental curves for the UTR2αsh and UTR2αint are reported in Supplementary Figure S5.

To evaluate whether the binding of the RNA moieties affects SHMT1 enzyme activity, we performed *in vitro* biochemical assays using the selected SHMT UTR sequences; yeast tRNA was used as a control of a RNA moiety unrelated to 5′UTRs. The serine hydroxymethyltransferase activity of the enzyme, whereby L-serine and THF are converted into glycine and 5,10-CH_2_-THF, was measured by incubating SHMT1 (0.2 μM) with a saturating concentration of serine (10 mM), 80 μM THF and varying concentrations of different RNAs. The ability of SHMT1 to catalyze this reaction was clearly inhibited by all the RNAs, although to different degrees (Figure 7 and Supplementary Figure S5). UTR2 showed the highest inhibitory efficacy (Figure 7A), whereas UTR1, UTR2αsh and tRNA had the lowest and UTR2αint had an intermediate inhibitory efficacy (Supplementary Figure S5). The estimated apparent inhibition constants (i.e. the RNA concentration required to obtain 50% activity inhibition, IC_50_) are summarized in Figure 7C.

Given that UTR2 shows the higher inhibitory efficacy, we then subdivided this fragment in shorter segments, to better identify which region of the UTR2 is mainly responsible for binding and inhibition of SHMT1. Three shorter UTR2 segments were produced and assayed: UTR2_1–100_, which comprises the first 100 nts, UTR2_101–206_, containing only the last 106 nts and a third segment lacking the first 50 nts (UTR2_51–206_ ). As shown in Figure 7B and C, the UTR2_1–100_ segment has a 4-fold higher inhibitory capacity as compared to the UTR2_101–206_ one, suggesting that the region of interest is likely located in the first half of UTR2. Interestingly, the third segment lacking only the first 50 nts (UTR2_51–206_ in Figure 7B) behaves in a similar way as the UTR2_101–206_ fragment. EMSA experiments confirm this trend (Supplementary Figure S6).

Altogether, these findings indicate that SHMT1 likely binds with high affinity to a sequence and/or a secondary structure motif contained in the first 50 nts of UTR2. Since, among all the assayed RNAs, the complete UTR2 still displays the highest binding and inhibition propensity, a synergistic contribution of the complete sequence is probably required for optimal SHMT1 binding.

The inhibitory effect of RNA on the reverse SHMT reaction was measured employing two parallel experimental approaches, i.e. following the conversion of glycine and 5,10-CH_2_-THF into L-serine and THF and measuring the formation of the quinonoid intermediate that develops when glycine and 5-CHO-THF-Glu bind to the enzyme forming a ternary complex (23). Surprisingly, a milder inhibitory effect of UTR2 was observed with both kinetic assays, even at the maximal RNA concentration employed (Supplementary Figure S7). In these experiments, UTR2 was the most efficient inhibitor with an IC_50_ of ≈4 μM. The formation of the quinonoid intermediate in the presence of UTRs was evaluated using both the mono- and pentaglutamylated form of the folate substrate but no significant differences were observed (data not shown).

In agreement with the results obtained by EMSA analyses, the activity of the K157S/K158S mutant was unaffected by UTR2, the most efficient inhibitor of the wild-type protein (Supplementary Figure S8).

### Mechanism of SHMT inhibition by RNA

**Figure 8.**
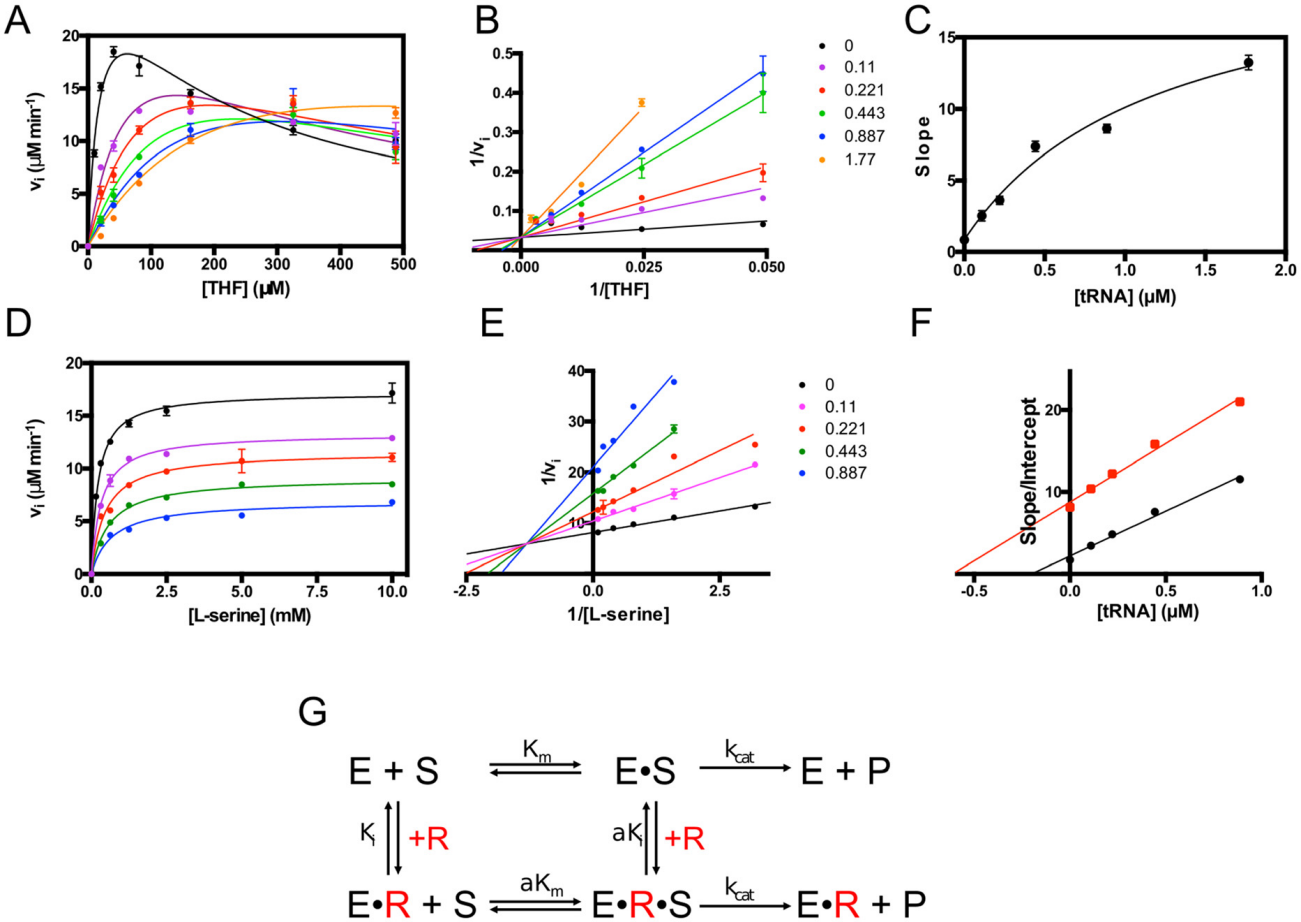
Mechanism of inhibition of SHMT1 catalysis by RNA. (**A**) Initial velocity of the SHMT reaction was measured at different THF concentrations, in the presence of saturating L-serine (10 mM) and fixed concentrations of tRNA: 0, 0.11, 0.22, 0.44, 0.88 and 1.77 μM. The continuous lines through the experimental points (which are the average of three independent experiments) are those obtained upon the least square fitting of data to Equation (3) (see ‘Materials and Methods’ section). (**B**) Double reciprocal plots of experimental points from A, after exclusion of the substrate inhibition portion of data. The continuous straight lines through the experimental points were obtained from the independent linear fitting of data. (**C**) Dependence of the slope, calculated in double reciprocal plot (B), on tRNA concentration. The continuous line through the experimental points was obtained from fitting to a saturation curve. (**D**) Initial velocity of the SHMT reaction was measured at different L-serine concentrations, in the presence of 80 μM THF and fixed concentrations of tRNA: 0, 0.11, 0.22, 0.44, 0.88 and 1.77 μM. The continuous lines through the experimental points (average of three independent experiments) are those obtained upon the least square fitting of data to Michaelis–Menten equation. (**E**) Double reciprocal plots of experimental points from D. The continuous straight lines through the experimental points were obtained from the independent linear fitting of data. (**F**) Replots of data taken from the double reciprocal plot (E): slope (*K*_m_/*V*_max_; in black) and intercept (1/*V*_max_; in red) versus tRNA concentration. The continuous straight lines through the experimental points were obtained from linear fitting. (**G**) Equilibria describing hyperbolic competitive inhibition between SHMT1 and RNA (data shown in panels A–C). In this particular case, a saturating amino acid substrate concentration (either L-serine or glycine) is present and therefore E corresponds to the enzyme–amino acid complex. E•S is the ternary complex with the folate substrate (either THF or CH_2_THF). If RNA (R) is present, it binds to both E and E•S forming E•R and E•R•S complexes, respectively.

To characterize the molecular mechanism of the RNA-mediated inhibition, we performed a detailed study of SHMT1 inhibition kinetics. Since tRNA inhibits the enzyme’s activity (Figure 7A), although less efficiently, and is easily available in the large amounts necessary to perform these experiments, we employed tRNA instead of UTR2 for the mechanistic studies. As a first step, the initial velocity of the reaction was measured at different tRNA concentrations, keeping L-serine concentration fixed (10 mM) and varying the THF concentration. Results show a typical THF substrate inhibition (33) (Figure 8A), which became less evident at increasing tRNA concentrations. Fitting of data to Equation (3) (see ‘Materials and Methods’ section), a modified Michaelis–Menten equation that accounts for substrate inhibition, showed that while tRNA increased the *K*_m_ for THF, it did not affect *V*_max_. After exclusion of the substrate inhibition portion of data, a double reciprocal plot confirmed this observation, which is typical of competitive inhibition (Figure 8B). However, slopes (*K*_m_/*V*_max_) obtained by fitting the data in the double reciprocal plot did not show the characteristic linear dependency on inhibitor concentration, and instead exhibited a hyperbolic behaviour (Figure 8C). Moreover, plots of initial velocity measured at a fixed THF concentration as a function of tRNA clearly showed that the limiting velocity as the inhibitor concentration tends to infinity was not zero (Figure 7A). All these characteristics indicate that tRNA is not a pure competitive inhibitor and suggest a ‘partial competitive’ or ‘hyperbolic’ inhibition (34). In this particular type of inhibition, the substrate and the inhibitor bind to partially overlapping or totally different sites and have higher affinity for the free enzyme than for the enzyme-inhibitor and enzyme–substrate complexes, respectively. When the enzyme forms a complex with one ligand, the affinity for the other ligand is decreased by an equal *α* factor (Figure 8G). Nevertheless, the enzyme-substrate and the enzyme–substrate–inhibitor complexes yielded the product with equal rate constants. Global fitting of velocity data to Equation (4) (see ‘Materials and Methods’ section), which combines hyperbolic competitive inhibition to substrate inhibition, gave estimates of K_i_ for tRNA (0.031 ± 0.005 μM) and of *α* factor (18.8 ± 4.6). It is noteworthy that, at the highest THF concentrations used in the experiments, tRNA attenuated THF substrate inhibition (Figure 8A). This paradoxical situation is caused by the decrease of affinity for the folate substrate induced by tRNA.

In a second set of experiments, the initial velocity in the presence of increasing tRNA was measured at a fixed THF concentration (0.08 mM), while varying L-serine (Figure 8D). In this case, the double reciprocal plot of data (Figure 8E) and the behaviour of the obtained slopes and intercepts (Figure 8F) indicated a pure mixed inhibition, showing that tRNA mainly competes with folate but not with L-serine.

### UTR2 modulates cellular SHMT1 activity and cell viability

**Figure 9.**
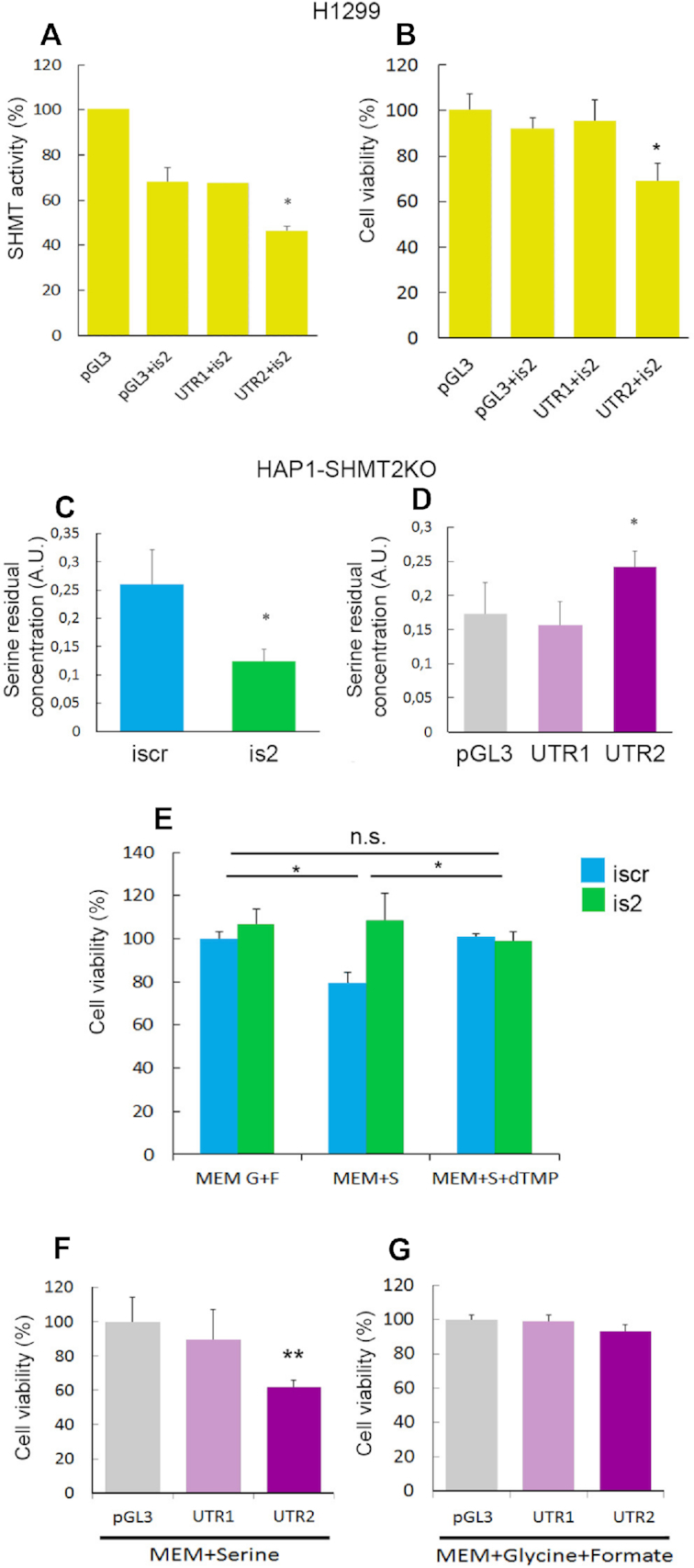
SHMT activity and cell viability in H1299 and HAP1-SHMT2KO cells. (**A**) SHMT enzymatic activity in H1299 cells 48 h after transfection with the indicated plasmids (pGL3 alone or with UTR1 and UTR2, respectively) and iRNAs measured by tritium-exchange radioisotopic assay. (**B**) Cell viability evaluated by Trypan blue exclusion assay in H1299 cells cultured in MEM supplemented for 24 h with 0.4 mM serine the day after transfection with the indicated iRNAs and/or plasmids. SHMT1 activity in HAP1-SHMT2KO cells assessed evaluating serine residual concentration in the supernatants 72 h after transfection with the indicated RNAis (**C**) or plasmids (**D**). (**E**) Cell viability evaluated in HAP1-SHMT2KO cells cultured in MEM supplemented for 48 h with 0.4 mM glycine (G) + 0.5 mM formate (F), 0.4 mM serine (S) and/or 30 μM thymidine 5′monophosphate (dTMP) the day after transfection with the indicated RNAis (E) or plasmids (**F** and **G**). Statistical analysis are performed on three independent experiments * *P* ≤ 0.05.

To test the effect of UTR2 on SHMT1 activity in the cells, we first used the H1299 lung cancer cell line (13). The SHMT activity was measured in intact cells using a technique optimized by our group (25). In order to better evaluate the SHMT1 contribution to this activity, we have co-transfected the H1299 cells with ishmt2, thereby silencing SHMT2 protein expression, and with plasmids containing either UTR1, UTR2 or a control plasmid (Figure 9A). Under these conditions, the SHMT1 activity was ∼25% lower in the cells overexpressing UTR2 with respect to those transfected with the plasmid containing UTR1 or the control plasmid. In parallel, we also evaluated cell viability (Figure 9B). The cells were co-transfected with ishmt2 together with UTR2 (or UTR1) expressing plasmid or pGL3 and maintained in MEM supplemented with serine. In this cellular model, UTR2 overexpression decreased cell viability by 35%; this nicely parallels the observed decrease in SHMT activity, suggesting that the two parameters are likely correlated.

We evaluated SHMT1 activity also in HAP1 cells, a human haploid cell line previously used as a valid metabolic model for 1C-metabolism (21,35,36), by measuring the consumption of exogenous serine. We recently reported that SHMT2 knockout (HAP1-SHMT2KO) cells can survive and proliferate in MEM supplemented with serine, suggesting that SHMT1 in the HAP1-SHMT2KO cells is able to perform the serine to glycine reaction, thereby complementing the SHMT2 deficiency (21). Of note, the HAP1-SHMT2KO cells were produced by a CRISPR/CAS9-mediated frameshift of shmt2 mRNA in exon 2, and thus the SHMT2 protein is not produced (as confirmed by experiments shown in Figure S9), but the mRNA, including the UTR2 portion, is still expressed. To eliminate the possible SHMT1 inhibition, which might arise from UTR2-containing mRNA still present in the SHMT2KO cells, we transfected the cells with ishmt2 (or iscr as control), prior to analysis. At 24 h after transfection, the complete medium was exchanged for MEM supplemented with 400 μM serine. We then measured serine consumption using gas chromatography/mass spectrometry, by evaluating the residual concentration of serine in the conditioned media of the cells 48 h after medium change. As shown in Figure 9C, HAP1-SHMT2KO cells transfected with ishmt2 consumed twice as much serine as the control cells (transfected with iscr); thus, serine consumption is modulated by the presence of UTR2-RNA, an effect which can be due to UTR2 binding to SHMT1.

In a parallel experiment, the HAP1-SHMT2KO cells were transfected with pGL3 alone, UTR1 or UTR2-containing vectors and serine was measured as described above. In these experiments, according to our working hypothesis, overexpression of UTR2 should affect SHMT1-driven serine consumption. Confirming our prediction, residual serine in the medium was 40% higher in the cultures transfected with UTR2 than in cells transfected with UTR1 or the control plasmid pGL3 (Figure 9D). These results highlighted that the biological effect was UTR2-specific.

To analyse whether the UTR2-containing mRNA affects cell viability, we transfected HAP1-SHMT2KO cells with either ishmt2 or iscr, and then cultured the cells in MEM supplemented with serine or glycine + formate and evaluated the number of live cells. As mentioned above, transfection with ishmt2 should eliminate the SHMT1 inhibition, which might arise from UTR2-containing mRNA still present in the SHMT2KO cells. We found a ∼35% increase in ishmt2-transfected cell viability over equivalent iscr-transfected cells only when MEM was supplemented with serine (Figure 9E). To further show that cell viability is related to SHMT1 activity, we have supplemented MEM with dTMP, a metabolite whose synthesis requires the CH_2_-THF produced in the serine to glycine breakdown mainly catalyzed by SHMT1 (11). Consistent with our hypothesis, dTMP supplementation led to similar cell viability in both iscr- and ishmt2-transfected cells (Figure 9E).

Finally, to assess if the overexpression of UTR2 in SHMT2-KO cells could also affect cell viability, the cells were transfected with a pGL3 plasmid with or without UTR2 and UTR1. Trypan blue exclusion assay demonstrated that UTR2 overexpression induced a decrease in cell viability of ∼40%, but only when cells were grown in MEM supplemented with serine, an effect that was not observed with UTR1 (Figure 9 F and G).

## DISCUSSION

Cell proliferation is necessarily a tightly regulated mechanism and 1C-metabolism is at the heart of the production of precursors necessary for proliferation. SHMT proteins are central players in 1C-metabolism in normal and cancer cells (37), as is evident by the recent focus on these enzymes in the literature (37,38). 1C-metabolism is compartmentalized with dedicated cytosolic (SHMT1) and mitochondrial (SHMT2) enzymes and the exchange of metabolites was shown to link the two compartments (5). Previous evidence has shown that SHMT1 is able to shift its activity to produce serine from glycine or vice versa, depending on the metabolic needs of the cells, thus supporting 1C-metabolism (5). The present study reports a novel regulatory mechanism that depends on the moonlighting property of cytosolic SHMT1 to bind specific RNA molecules and suggests that the formation of the SHMT1–RNA complex has far-reaching consequences, as it is able to fine-tune SHMT2 expression and, at the same time, to modulate the enzymatic activity of SHMT1.

We showed that human SHMT1 is able to bind *in vitro* to SHMT2 5′UTR sequences and provided evidence to suggest that this interaction might cooperate to control cellular SHMT2 mRNA and protein levels. SHMT isozymes have been previously suggested to be potential RNA-binding proteins (39) and *in vitro* experiments indicated that SHMT1 is capable of autogenously regulate its expression by binding to the 5′UTR of its mRNA, in a similar manner to that reported for TYMS and DHFR, the other two enzymes of the thymidylate cycle (2,3,15). This RNA-binding property of SHMT is evolutionary conserved, as it was also observed in *Plasmodium* SHMT (40). The RNAs identified in the present study (SHMT2 5′UTRs) exhibit a high affinity for the SHMT1 protein. More importantly, our observations on the UTR2 RNA allow us to propose a novel regulatory mechanism connecting the cytosolic and mitochondrial SHMT-dependent 1C-metabolism.

Metabolites, including SHMT substrates, play an important role in the cross-talk between the cytosolic and mitochondrial compartments of 1C-metabolism. Here, we addressed whether and how SHMT-related metabolites might also affect the formation of the RNA/protein complex. Different molecular mechanisms, such as mutually exclusive binding to the same protein region, allosteric control or even control of metabolite-driven post-translational modifications could explain the effect of metabolites on the protein–RNA complex formation (1). We show here that serine and glycine alone are not sufficient to perturb the formation of the SHMT1–RNA complex *in vitro*, whereas the pentaglutamylated folate competes with UTR2 for binding to SHMT1, an effect that is enhanced by increasing the glycine concentration. Our results thus support the idea that glycine and folate(s) modulate the formation of the SHMT1–RNA complex. It should be underscored here that the amounts of 5-CHO-THF-Glu_5_ and glycine necessary to compete for the *in vitro* binding of UTR2 fall within the physiological range (41). Moreover, based on our results with penta- and mono-glutamylated species in the presence of glycine, we predict that also polyglutamylated folates, the predominant form of folates in all organisms (42,43), can be effective competitors of RNA binding in the cell.

The competition between folates and RNA, together with the findings from the site-directed mutants and the kinetic characterization showing hyperbolic inhibition behaviour, strongly suggest that the RNA-binding site on SHMT1 involves the folate main binding cleft. However, the observed difference in binding between the mono- and penta-glutamylated folate species highlights that the polyGlu tail is critical in determining binding propensity, suggesting that the RNA-binding site likely extends beyond the substrate binding site, involving the positively charged residues that surround the folate binding cleft (Figure 6F). We thus believe that this region can be considered a non-canonical RNA-binding site. Interestingly, binding of selenocysteinyl tRNA (tRNA^Sec^) by human O-phosphoseryl-tRNA:selenocysteinyl-tRNA synthase (SepSecS), the enzyme that catalyzes the final step of selenocysteine synthesis, also occurs through a similar interface (44). SepSecS belongs to the same family of fold-type I pyridoxal phosphate enzymes that include SHMT (44–46) and has a quaternary structure closely resembling that of human SHMT. The conservation of the non-canonical RNA-binding site across different members of this fold type may suggest that other proteins of this family could be involved in RNA binding, possibly regulating other cellular processes.

Interestingly, the formation of the metabolite-sensitive RNA–protein complex also exerts an RNA-mediated control on SHMT1 enzymatic activity. The cleavage of serine is decreased in the presence of RNA, with UTR2 appearing as the strongest inhibitor (IC_50_ ∼33 nM). The notion that the competition between an enzyme’s cofactor/substrate and RNA can affect the catalytic activity is already known for other metabolic enzymes, including GAPDH (47). On the other hand, the discovery that the RNA-mediated inhibition can act selectively is unprecedented, as has not previously been observed for SHMT or other metabolic enzymes. Our results show for the first time that, whereas RNA binding inhibits serine cleavage, unexpectedly the reverse reaction (SHMT1-driven conversion of glycine into serine) is much less affected. Binding of RNA to the SHMT–serine complex somehow hampers the binding of the folate substrate more than binding of RNA to the SHMT–glycine complex. A possible mechanistic interpretation is illustrated in the scheme shown in Figure 8G, assuming the value of the *α* factor to be higher in the forward SHMT reaction (serine to glycine direction) than in the reverse SHMT reaction (glycine to serine direction). It is known that the SHMT-serine and SHMT–glycine complexes are in a different conformational status; in particular L-serine binding induces an open-to-close conformational change that rearranges the protein region close to the active site. This change that does not occur upon binding of glycine (48). Therefore, it is plausible that the interaction of RNA with such dissimilar conformations of the enzyme may differentially affect the binding of the folate substrate and the subsequent catalytic steps. Accordingly, SHMT small molecule inhibitors show a similar behaviour, mainly affecting the hydroxymethyltransferase reaction when serine acts as a substrate (21,49). The *in vitro* results presented here on UTR2 segments (Figure 7B) strongly suggest that it is possible to identify shorter constructs capable of maintaining a reasonable affinity and inhibition potential for SHMT, a proof-of-concept of the feasibility of designing nucleic acid-based SHMT inhibitors.

The observed RNA-mediated inhibition of SHMT1 characterized *in vitro* was validated using SHMT2-knockdown and null cell lines. First, UTR2 (but not UTR1) RNA lowers the SHMT intracellular activity, highlighting the specificity of UTR2-mediated inhibition. Second, in a SHMT2-null background, serine consumption increases when UTR2 is removed, whereas its overexpression leads to an accumulation of this amino acid in the medium, suggesting that the serine to glycine reaction is inhibited. Finally, the inhibition of SHMT1 by UTR2 also correlates with a significant reduction of cell viability in serine-containing medium. These data suggest that in these models, in the absence of SHMT2, when the hydroxymethyltransferase reaction is catalyzed by the cytosolic isozyme, a decrease of cell viability occurs, which is likely due to reduced serine consumption by SHMT1 following the formation of the protein–RNA complex.

Future analysis will be needed to fully elucidate the structural features of the non-canonical RNA-binding domain of SHMT1, as well as of its complexes with RNA, and to assess the effectiveness in the cells of novel nucleic acid-based inhibitors of the SHMT enzyme, which represents an attractive target for novel metabolic anti-tumour chemotherapy.

Our study adds a novel and thus far unpredicted layer of complexity to the regulation of SHMT expression and activity. SHMT was already known to be controlled at both transcriptional (50) and post-translational level (51) and a cross-talk between SHMT1 and SHMT2 expression has also been reported (5,13). Nevertheless, the molecular mechanisms by which one protein could affect the expression of the other isoform were unclear. In our cellular models, the RNA-binding activity of SHMT1 plays an important role, controlling SHMT2 expression, at least in part, at the translational level. In the working hypothesis presented here, when SHMT2 mRNA is highly expressed, the cytosolic enzyme SHMT1 likely binds to it and the glycine to serine conversion by the cytosolic enzyme is favoured. When SHMT2 levels are reduced, the SHMT1 enzyme can work in the opposite direction, as the RNA-mediated inhibition is relieved. In parallel, reducing SHMT1 protein levels may affect SHMT2 mRNA levels available for translation, thus favouring SHMT2 expression. Other metabolic parameters, such as the distribution and levels of metabolites (folate species, glycine and indirectly possibly also NADH/NADPH) might also contribute to this RNA-mediated regulatory mechanism. Altogether, this strategy may contribute to maintain the directionality of the SHMT1 reaction towards serine synthesis, also allowing the intracellular serine to be metabolized in the mitochondria. Although we are aware that the specific traits of this regulation may vary in different cellular milieus, we noticed that UTR2-containing transcripts are also highly expressed in other tissues (Tables S5-S7 for colon, liver and kidney), thus it would be worth to investigate if the cross-regulation mechanism presented here is active also in these histotypes.

Finally, since downregulation of SHMT1 was found associated with the modulation of the expression of several genes (52), we believe that our findings are just a small part of a broader phenomenon in which SHMT1 (53,54), as frequently observed for RNA-binding proteins (55), potentially regulates the expression of multiple genes at the post-transcriptional level. Future studies will attempt to characterize in-depth the regulatory role of the cytosolic SHMT, to date unexplored. Furthermore, the relevance of the RNA-binding activity of mitochondrial SHMT will also need to be addressed.

Overall, the metabolite-RNA competition events shown in this work fit well within the general model by which alterations in cellular metabolism are sensed by metabolic enzymes via RNA (or vice versa) as suggested in the so-called ‘REM (RNA–enzyme–metabolite) hypothesis' (56). This scenario is becoming increasingly more important as the expanding fields of metabolic studies and RNA complexity controlling cell function begin to intersect.

## DATA AVAILABILITY

UCSC Genome Browser is available at https://genome.ucsc.edu/.

omiXcore is freely accessed on the web at http://service.tartaglialab.com/grant_submission/omixcore.

Global Score is available at http://service.tartaglialab.com/new_submission/globalscore.

## Supplementary Material

Supplementary Data
