# Supplementary material for: The moonlighting RNA-binding activity of cytosolic serine hydroxymethyltransferase contributes to control compartmentalization of serine metabolism
Source: Nucleic Acids Res. 2019 Feb 27;47(8):4240–54. doi: 10.1093/nar/gkz129 (PMC6486632; doi:10.1093/nar/gkz129)
Supplement: Supplementary Data [file gkz129_supplemental_files.zip › Guiducci-Supp-revised-FINALE.pdf]

Supplementary Information

The moonlighting RNA-binding activity of cytosolic serine hydroxymethyltransferase contributes to control compartmentalization of serine metabolism

Guiducci et al.

Supplementary Materials and Methods

SHMT1 and SHMT2 isoforms expression analysis RNA-seq data (level 3, RNA-seq v2 expression data) were obtained from The Cancer Genome Atlas - Lung Adenocarcinoma (TCGA-LUAD; tumor samples: 516; normal controls: 59; (1)). The estimated transcript quantification from RNA-Seq data of SHMT1 and SHMT2 transcripts was computed using RSEM (2). Median transcripts per million (TPM) was computed by counting up all the reads per kilobase values in a sample and dividing this number per million scaling factor. UCSC (hg19/GRCh37; https://genome.ucsc.edu/) was used for isoforms definitions.

***In vitro* translation** Translation was measured as the synthesis of luciferase protein from the corresponding mRNAs using Flexi Rabbit Reticulocytes Lysate System kit (Promega). The RNAs employed in the *in vitro* translation assays were produced from pGL3 plasmids as described in the *in vitro* transcription section of main text. The luciferase mRNA with or without the upstream UTR2 sequence was heated 3 minutes at 65 °C and immediately cooled down in an ice-water bath. The transcripts were incubated 5 minutes at RT with SHMT1 in presence of recombinant Rnasin Ribonuclease Inhibitor (40 U); the other reaction components were then added to obtain a final mixture containing 125 ng RNA, 0.6 μM SHMT1, 17.5 μl rabbit reticulocyte lysate, 20 μM amino acid mixture and 70 mM KCl. The translation reactions were incubated 30 minutes at 30 °C and then stopped in ice. Luminescence was detected according to the manufacturer's protocol using VICTOR™ Multilabel Counter (Perkin Elmer, USA) equipped with 96-well plate reader.

Supplementary References

1. Tomczak,K., Czerwińska,P. and Wiznerowicz,M. (2015) The Cancer Genome Atlas (TCGA): an immeasurable source of knowledge. *Contemp. Oncol. (Poznan, Poland)*, **19**, A68-77.
2. Li,B. and Dewey,C.N. (2011) RSEM: accurate transcript quantification from RNA-Seq data with or without a reference genome. *BMC Bioinformatics*, **12**, 323.

Supplemental Table S1. Nucleotide sequences of the primers used in the studies (written 5' to 3'). Forward primers used to amplify the constructs employed in the *in vitro* transcription experiments contain the T7 promoter sequence at the 5' terminal (underlined in the text).

**RNA**	***In vitro* Transcription**
UTR1	Fw taatacgactcactatagggcctggcgcgcagagtgcacc<br>Rv tgcactggttcgaagctgcc
UTR2	Fw taatacgactcactatagggataaagaaaaaagcggtgag<br>Rv cgcaactcggaagtcgcaggag
UTR2α short	Fw taatacgactcactatagggacggactgctaaaggtctcccc<br>Rv cctgaccagctgcccacatctctg
UTR2α intermediate	Fw taatacgactcactatagggtttcacgtggcattaggggag<br>Rv cctgaccagctgcccacatctctg
$UTR2_{1-100}$	Fw taatacgactcactatagggataaagaaaaaagcggtgag<br>Rv ggcgccaaagctaacgggag
$UTR2_{101-206}$	Fw taatacgactcactatagggtcagcgagctcttctcgcgc<br>Rv cgcaactcggaagtcgcaggag
$UTR2_{51-206}$	Fw taatacgactcactatagggaaggggccaccactacgcatgc<br>Rv cgcaactcggaagtcgcaggag
luc	Fw taatacgactcactatagggatggaagacgccaaaaacataaag<br>Rv gtggtttgtccaaactcatc
UTR2-luc	Fw taatacgactcactatagggataaagaaaaaagcggtgag<br>Rv gtggtttgtccaaactcatc
**Mutant**	**Site-Directed Mutagenesis**
SHMT1 K157S/K158S	Fw cccatgggttcatgacagacagcagcaaaatctctgccacgtccatc<br>Rv gatggacgtggcagagattttgctgctgtctgtcatgaacccatggg
SHMT1 Y82A	Fw ggtacccgggccagagagcctatggcgggact<br>Rv agtcccgccataggctctctggcccgggtacc
**Sample**	**Real-Time PCR**
SHMT1	Fw aggaaaggagtgaaaagtgtggat<br>Rv gacaccagtgtcgctctggatctg
UTR2	Fw ggcgaactacaattcccaaa<br>Rv actctggtctagggcagcaa
β-ACTIN	Fw aggatggcaagggacttcctg<br>Rv aatgtggccgaggactttgat

Supplemental Table S2. The enclosed Excel file reports the complete list of the binding potentials and the binding affinities for the 164 sequences having the same length as the SHMT2 5'UTR (UTR2) sequence calculated using *Global Score* and *omiXcore*.

Supplemental Table S3. Expression of the SHMT1 and SHMT2 transcripts selected for this study in Normal (N) and LUAD (T) cells.

Isoforms*	*TPM*		*Percentage*	
	Normal	*Tumor*	*Normal*	*Tumor*
<u>SHMT1</u> *(Gene: ENSG00000176974)*				
NM_004169[a] (*uc002gta.3*) 5'UTR 191nt	*13.9*	*14.8*	*73.9%*	*67.9%*
NM_148918[a] (*uc002gtb.3*) 5'UTR 191nt	*3.2*	*4.8*	*17.1%*	*22.1%*
<u>SHMT2</u> *(Gene: ENSG00000182199)*				
NM_005412 (*uc001snf.2*) 5'UTR 205nt	*26.5*	*63.1*	*95.9%*	*92.5%*
NM_001166358 (*uc001sni.2*) 5'UTR 230nt	*0.3*	*2.4*	*1.1%*	*3.5%*
NM_001166359 (*uc010srh.2*) 5'UTR 123nt	*<0.01*	*<0.01*	-	-

TPM: Transcripts-per-million.

[a] The two SHMT1 transcripts share the same UTR.

*<u>Minor SHMT1 isoforms</u> - **uc002gtc.1**:N, 0.2; 1.3% / T, 0.5; 2.0% - **uc002gtd.1**:N, 0.9; 5% / T, 0.8; 3.9% - **uc002gsz.3**:N, 0.4; 2.1% / T, 0.7; 3.4% -**uc010vxu.1**:N, 0.1; 0.6% / T, 0.1; 0.6%.

<u>Other minor SHMT2 isoforms -</u> **uc001snj.2**:N, 0.15; 0.6% / T, 0.7; 1.1% - **uc010srj.2**:N, -; - / T, 1.2; 1.8% - **uc001snh.2**: N, 0.7; 2.5% / T, 0.8; 1.1%.

Supplemental Table S4. Nucleotide sequence of the 5'UTR RNAs used in the studies.

5'UTR	RNA sequence (5' to 3')
UTR1	gccuggcgcgcagagugcaccuuccugagcucgagcgguccagcgccaaguucgggguuuggggguuggagcggcuggucacguggcuggcccgcg<br>gcggugcgcggggcguugggucagcgggucugggacugguggcaccggcggcggcguaggacggaggcgucgcuaggcagcuucgaaccagugca
UTR2	auaaagaaaaaagcggugagugggcgaacuacaauucccaaaaggccacaaaggggccaccacuacgcaugcguagaucccucccguuagcuuuggcgccuca<br>gcgagcucuucucgcgcaugcguucuccgaacggucuucuuccgacagcuugcugcccuagaccagaguugguggcuggaccuccugcgacuuccgaguugcg
UTR2α short	acggacugcuaaaggucuccccucccaccugcauugcucuacaaucuguggccucagcccu<br>ugccaacucugccucuucccccgcugacugggccucugcagagaugugggcagcuggucagg
UTR2α intermediate	uuucacguggcauuaggggagaggacagccccggaugccccgcggacccggugcuggcaaaugagcggaguuuucggccuggucucacaagcugagccuuu<br>ccggccagcucugagcccugcagaugaugcaaccgagcuucacugcuugcaucaggcaggggucccggcggcagugagggcuggaaggagguggaacggcc<br>ucugcagagaugugggcagcuggucagg

Supplemental Table S5. Expression of SHMT1 and SHMT2 isoforms in normal (N) and colon adenocarcinoma (T) cells.

Isoforms	*TPM*		*Percentage*	
	Normal	*Tumor*	*Normal*	*Tumor*
SHMT1 *(Gene: ENSG00000176974)*				
NM_004169 (*uc002gta.3*) 5'utr 191nt	*18.4*	*14.9*	*69.7%*	*66.4%*
NM_148918 (*uc002gtb.3*) 5'utr 191nt	*5.2*	*5.5*	*19.9%*	*24.5%*
SHMT2 *(Gene: ENSG00000182199)*				
NM_005412 (*uc001snf.2*) 5'utr 205nt	*46.7*	*112.2*	*95.6%*	*94.5%*
NM_001166358 (*uc001sni.2*) 5'utr 230nt	*1.0*	*2.1*	*1.5%*	*1.3%*
NM_001166359 (*uc010srh.2*) 5'utr 123nt	*<0.01*	*<0.01*	-	-

TPM: Transcripts-per-million

Supplemental Table S6. Expression of SHMT1 and SHMT2 isoforms in normal (N) and liver hepatocellular carcinoma (T) cells.

Isoforms	*TPM*		*Percentage*	
	Normal	*Tumor*	*Normal*	*Tumor*
SHMT1 *(Gene: ENSG00000176974)*				
NM_004169<br>(*uc002gta.3*)<br>5'utr 191nt	*151.4*	*68.9*	*74.2%*	*71.0%*
NM_148918<br>(*uc002gtb.3*)<br>5'utr 191nt	*31.8*	*18.5*	*15.7%*	*19.5%*
SHMT2 *(Gene: ENSG00000182199)*				
NM_005412<br>(*uc001snf.2*)<br>5'utr 205nt	*89.5*	*85.6*	*81.6%*	*85.0%*
NM_001166358<br>(*uc001sni.2*)<br>5'utr 230nt	*10.9*	*8.3*	*10.0%*	*8.2%*
NM_001166359<br>(*uc010srh.2*)<br>5'utr 123nt	*<0.01*	*<0.01*	-	-

TPM: Transcripts-per-million

Supplemental Table S7. Expression of SHMT1 and SHMT2 isoforms in normal (N) and kidney renal clear cell carcinoma (T) cells.

Isoforms	*TPM*		*Percentage*	
	Normal	*Tumor*	*Normal*	*Tumor*
SHMT1 *(Gene: ENSG00000176974)*				
NM_004169 (*uc002gta.3*) 5'utr 191nt	*132.4*	*69.0*	*74.5%*	*67.5%*
NM_148918 (*uc002gtb.3*) 5'utr 191nt	*28.5*	*19.4*	*16.0%*	*19.0%*
SHMT2 *(Gene: ENSG00000182199)*				
NM_005412 (*uc001snf.2*) 5'utr 205nt	*38.6*	*112.8*	*95.1%*	*73.2%*
NM_001166358 (*uc001sni.2*) 5'utr 230nt	*1.0*	*26.6*	*2.4%*	*17.2%*
NM_001166359 (*uc010srh.2*) 5'utr 123nt	*<0.01*	*<0.01*	-	-

TPM: Transcripts-per-million

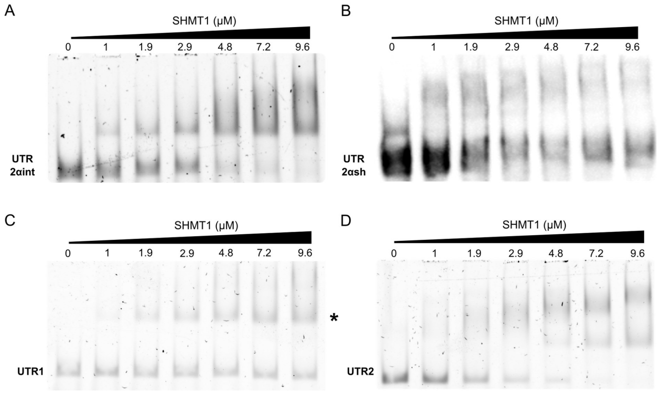

Supplemental Figure S1. Electrophoretic mobility shift assays. We confirmed the results shown in Fig. 2 using unlabeled 5'UTRs to exclude artifacts caused by the biotin moiety attached to the RNA molecules, also given that we observed that the biotinylation of the UTR2αint altered its affinity for SHMT1 (data not shown). We could not perform EMSA assay with unlabeled 5'UTR2αsh since it comigrates with tRNA. Migration of 0.24 µM unlabeled UTR2αint (A), UTR1 (C), UTR2 (D) and of 0.08 µM biotin-labeled UTR2α short (B) in the presence of the indicated concentrations of SHMT1. Densitometric measurements of the free RNA bands confirm the binding of SHMT1 to UTR2, UTR2αint and UTR2αsh and, in agreement with the results obtained with labeled RNA (Fig. 3B), shows that UTR1 is not bound by the protein. The protein-RNA complex visualized in (C), highlighted with a star (*), are composed of SHMT1 and tRNA, which is added in the reaction mixture as nonspecific competitor; densitometric analysis of the UTR1 confirms that the unbound does not vary significantly upon increasing SHMT1 concentration.

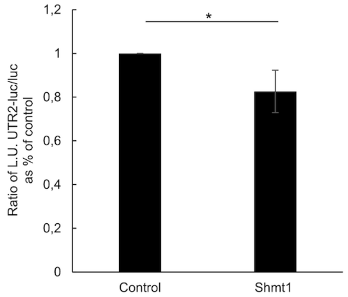

Supplemental Figure S2. Effect of SHMT1 on *in vitro* translation of UTR2-containing transcripts. Translation of luciferase mRNA with (UTR2-luc) or without (luc) the upstream UTR2 sequence was measured in the absence (Control) or presence (Shmt1) of 0.6 µM SHMT1. The results are plotted as the ratio of luminescence units (L.U.) between UTR2-luc and luc samples normalized on control reactions carried out in absence of SHMT1. Statistical analysis is performed on five independent experiments (* $P \leq 0.05$). Paired samples were analyzed with Student's t-test; all the others statistical analysis were performed using one way ANOVA followed by the Bonferroni post-hoc comparison test. A $p<0.05$ was considered significant.

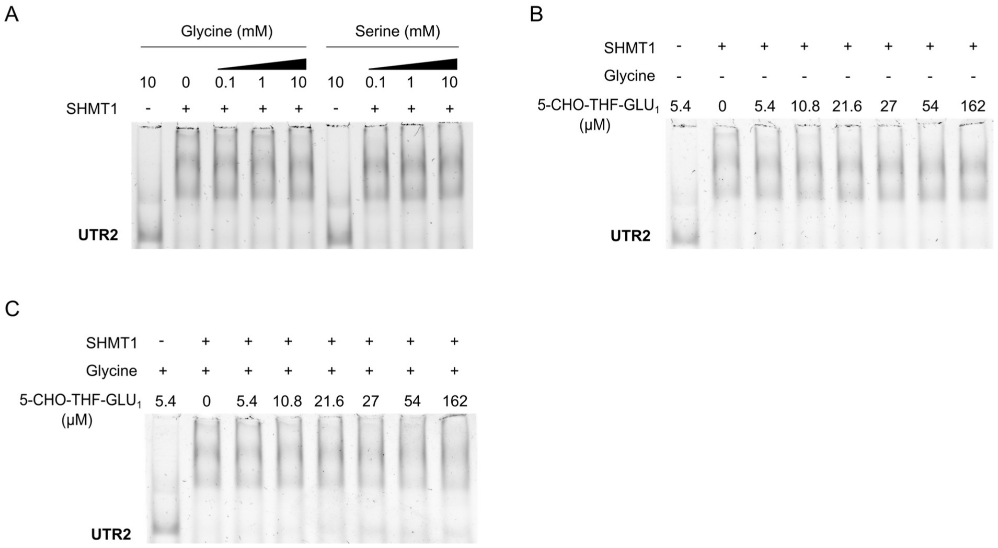

Supplemental Figure S3. Effect of SHMT substrates on UTR2 binding to SHMT1. EMSA carried out by incubating 5.4 µM of SHMT1 with the indicated amounts of 5-CHO-THF-Glu$_1$ in absence (A) or presence (B) of 10 mM glycine and with only serine or glycine (C) prior to the addition of 0.18 µM unlabeled UTR2.

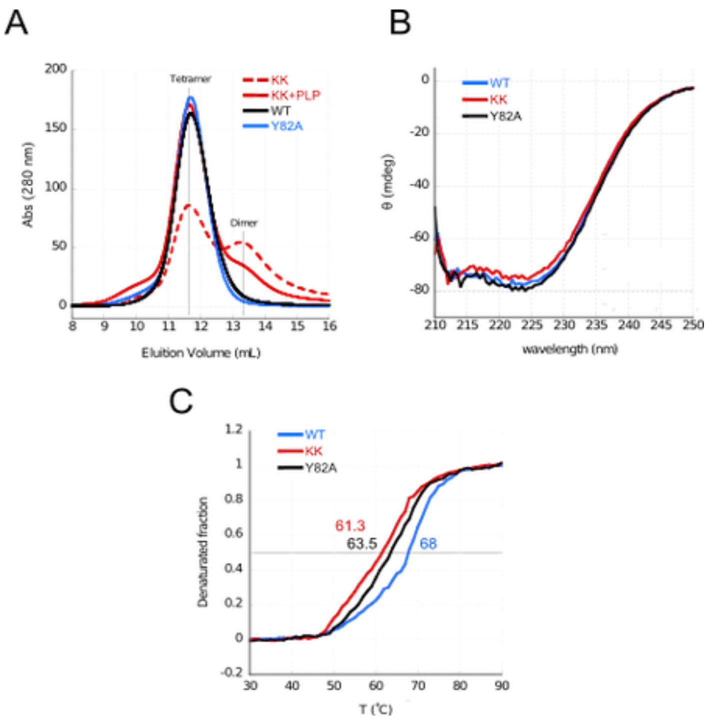

Supplementary Figure S4. Oligomerization state and stability of SHMT1 mutants. (A) Size exclusion chromatography profiles of wild type and mutant SHMT1 protein samples. The WT enzyme (*blue*) and the Y82A (*black*) mutant eluted as tetramers. In the same buffer (100 mM NaCl, 10 mM Hepes pH 7.4, 2.5% glycerol) the K157S/K158S mutant eluted as a mixture of dimer and tetramer (*dashed red line*). The tetrameric profile of this mutant was restored when 200 μM PLP is added to the eluition buffer (*continuous red line*). For all samples 500 μL of a 100 μM protein solution was loaded into a Superdex 200 10/300 gel filtration column and eluted at a 0.9 mL/min flux.

(B) Dichroic spectra of SHMT1 WT and its mutants. Spectra were recorded on a JASCO J-710 spectropolarimeter, equipped with a Peltier temperature controller, with a 1 mm path length quartz cuvette (Hellma). Measurements were performed at 20°C. All samples contained 10 μM protein in 100 mM NaCl, 10 mM Hepes pH 7.4, 2.5% glycerol.

(C) Thermal denaturation of SHMT1 WT and its mutants. After recording the dichroic spectra the samples (containing 10 μM protein in 100 mM NaCl, 10 mM Hepes pH 7.4, 2.5% glycerol) were subjected to thermal denaturation. The dichroic signal at 222 nm was recorded as a function of temperature. The denaturation process was not reversible, since the proteins precipitate after denaturation; therefore the data may not be fitted and only apparent melting temperatures (corresponding to the T at which the fraction of denaturated protein is 0.5) and qualitative information can be extrapolated. The mutants appear to have a slightly lower stability with respect to the WT.

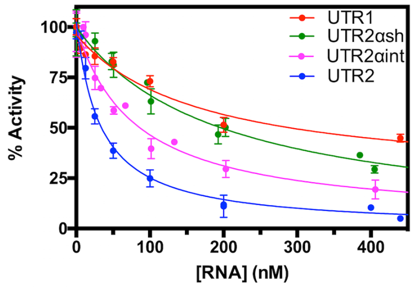

Supplemental Figure S5. Inactivation of SHMT1 by different RNAs. The inactivation of SHMT1 by UTR1 (in red), UTR2 (in blue), UTR2αsh (in green) and UTR2αint (in magenta), was followed by measuring the initial velocity of the hydroxymethyltransferase reaction, using L-serine and THF as substrates. Experimental data, which are the average of three independent experiments, were fitted to Eq.2 (see Methods) obtaining the continuous lines shown in the figure.

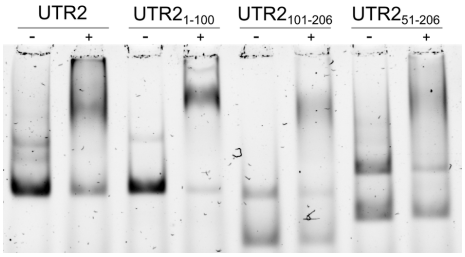

Supplementary Figure S6. Electrophoretic mobility shift assay on UTR2 segments. Migration of 0.5 μM unlabeled UTR2 and the indicated UTR2 segments in absence (-) or presence (+) of 4 μM SHMT1 (8-fold excess). The samples were run on a 4% polyacrylamide native gel.

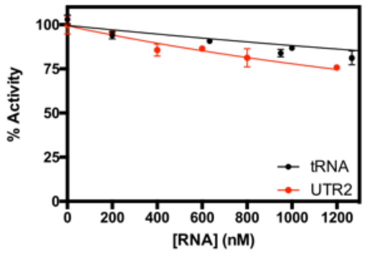

Supplemental Figure S7. Inactivation of SHMT1 by different RNAs in the reverse hydroxymethyltransferase reaction. The inactivation of SHMT1 by UTR2 (in red) and tRNA (in black) was followed as described in Methods. The figure combines the data obtained in three independent experiments with both assays described in the text, which were fitted to Eq. 2 (see Methods), obtaining the continuous lines showed in the figure.

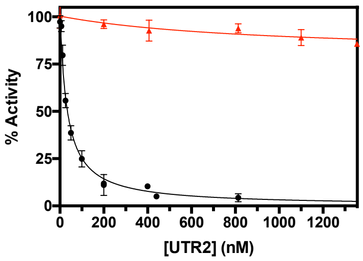

Supplemental Figure S8. Inactivation of wild-type and K157S-K158S mutant SHMT1 by UTR2 RNA. Kinetic parameters of the K157S-K158S SHMT1 mutant were determined (data not shown) and compared with those of the wild-type enzyme (Giardina et al. 2015, reference number 20 in the main text). While k_{cat} and K_m for serine were very similar for both enzyme forms, K_m for THF was about 5-fold larger in the case of mutant enzyme. Enzyme activity was followed by measuring the initial velocity of the hydroxymethyltransferase reaction using L-serine (10 mM) and THF (80 μM for wild type SHMT1 and 200 μM for the mutant enzyme) as substrates. The experimental data (black and red dots for the wild type and mutant SHMT1, respectively), obtained in three independent experiments, were fitted to Eq.2 (see Methods), obtaining the continuous lines shown in the figure.

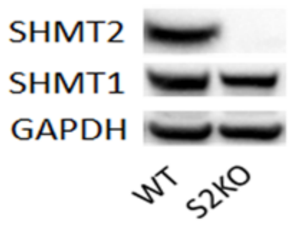

Supplemental Figure S9. Western blot analysis of SHMT1 and SHMT2 protein expression in HAP1 wild type (WT) and HAP1-SHMT2KO (S2KO) cells; glyceraldehyde 3-phosphate dehydrogenase (GAPDH) was used as loading control.
